# Assessment of large language model chatbots for hemodialysis meal planning: a descriptive study

**DOI:** 10.1186/s12882-026-04936-8

**Published:** 2026-03-31

**Authors:** Kevin Shi, Hiba Hamdan, Elizabeth Cheng, Delphine S. Tuot

**Affiliations:** 1 Division of Nephrology, Department of Medicine, University of California San Francisco, 500 Parnassus Avenue, MUW418 Box 0532, San Francisco, CA 94143 USA; 2 Division of Nephrology, Department of Medicine, University of California Davis, Davis, CA USA; 3 Department of Molecular and Cell Biology, University of California Berkeley, Berkeley, CA USA; 4 Center for Innovation in Access and Quality, Zuckerberg San Francisco General Hospital, San Francisco, CA USA

**Keywords:** Hemodialysis, Medical nutrition therapy, Large language models, Renal diet, Artificial intelligence

## Abstract

**Background:**

Large language models (LLMs) have the potential to improve nutritional counseling for patients with end stage kidney disease (ESKD). This study evaluates the utility of publicly available LLMs in generating meal plans for individuals receiving hemodialysis.

**Methods:**

Fifty hypothetical patient profiles were generated from United States national data and used to prompt four LLM chatbots, ChatGPT-o3-mini ^®^ (OpenAI), Claude Sonnet 3.7 ^®^ (Anthropic), Gemini 2.5 ^®^ (Google), and Llama 3.1 ^®^ (Meta), to create a single day meal plan accounting for ESKD dietary constraints. The primary outcome was concordance of LLM-generated meal plans with reference nutrition databases and specified nutrition goals. A secondary outcome was a qualitative usability assessment, as judged by three independent reviewers.

**Results:**

All models demonstrated substantial limitations in accurately representing nutrient content, particularly in underestimating phosphorus and potassium content in foods. Quantitatively, ChatGPT achieved the highest performance of the models studied with the highest average concordance with gold standard nutrition databases and with the lowest nutrient deviations from prompt goals. Gemini and Llama had less qualitative errors than ChatGPT and Claude, but all models frequently had vague outputs, often recommending composite foods without clear nutrient values.

**Conclusion:**

Currently, publicly available LLMs do not readily generate clinically acceptable meal plans for hemodialysis patients. All models misrepresented nutrient content and had significant usability concerns. Improvements in model architecture, knowledge bases, and domain-specific optimization will be required before LLMs can be safely used for dietary counseling for patients with ESKD.

**Supplementary Information:**

The online version contains supplementary material available at 10.1186/s12882-026-04936-8.

## Background

Precise dietary management is needed to maintain homeostasis for individuals with end stage kidney disease (ESKD). Without renal clearance of uremic toxins and modulation of fluid and electrolytes, individuals with ESKD are at risk for malnutrition, muscle wasting, volume overload, electrolyte abnormalities, cardiovascular complications, and bone disease. Optimal nutrition in ESKD requires management of numerous nutrients, including potassium, phosphorus, sodium, calcium, fiber, protein, acid load, and calories. For the over 3 million individuals on dialysis in the world [1], juggling the many dietary needs and constraints of ESKD is a difficult challenge, and patients must navigate a constellation of complex and often contradictory advice. For example, higher protein diets are preferred in ESKD to maintain body weight and prevent protein-energy wasting, but high protein foods are frequently also rich in phosphorus which can promote cardiovascular and bone disease [2]. Similarly, the low potassium diets recommended for patients with ESKD to avoid cardiac arrhythmias often recommend avoidance of fruits and vegetables [3], which are otherwise thought to have beneficial cardiovascular effects [4]. This is further complicated by systemic and personal factors that impact diet including food availability, neighborhood safety, income and socioeconomic status, occupation, personal schedule, medical comorbidities, cultural practices, and food preferences [5].

Medical nutrition therapy (MNT), or nutrition-focused assessment, treatment, and behavioral counseling, is a guideline-recommended facet of ESKD care [6]. In the United States, all patients who require dialysis are mandated to meet with a renal dietitian least monthly [7]. MNT, both with and without direct nutritional supplementation, has been linked to improved protein and calorie intake, nutritional and inflammatory markers, and electrolyte balance among individuals with ESKD [6, 8]. However, the efficacy of MNT is frequently limited by vague or contradictory advice, focus on individual lab metrics rather than understandable dietary patterns, patient and provider knowledge gaps, socioeconomic constraints, and lack of personalized, culturally sensitive advice to address the total patient food environment [9–13]. Only 31.5% of patients on hemodialysis are estimated to be able to follow a renal-restricted diet [14].

New large language models (LLMs) are well-poised to address this challenge. Artificial intelligence technologies have the capability to synthesize vast amounts of nutritional data and can potentially adjust recommended meal plans and dietary counseling to account for systemic and personal factors with precision and scope beyond what current methods offer. In addition, the traditional user-friendly conversational interface of LLMs can handle varied, customized inputs and offer users an opportunity to provide feedback on outputs. For example, LLMs can efficiently perform database look up to generate food substitutions upon request. Furthermore, new and upcoming features like on-the-spot translation and image generation may help with clarity of counseling. However, previous studies examining LLM performance in other nutritional contexts suggest that considerable improvements are required to LLMs before they can deliver clinically acceptable dietary counseling [15–17], with LLM-generated meal plans typically failing to meet critical macro- and micronutrient thresholds and improperly adjusting for allergies or patient-specific factors [18]. Given the increasing role of LLMs in daily life, a broader understanding of their capabilities and limitations in this domain is required before they could be incorporated as an adjunct to dietary counseling for patients with ESKD. This study evaluates the quantitative accuracy and subjective quality of four top-performing publicly available LLMs at providing patient-centered meal plans in the context of ESKD from February to April 2025.

## Methods

### LLM selection

Publicly available general purpose LLMs with high ratings in Multitask Reasoning and Math benchmarks were initially queried. Models with high accessibility, thorough documentation, and frequent updates were then selected for analysis: ChatGPT-o3-mini (OpenAI), Claude Sonnet 3.7 (Anthropic), Gemini 2.5 Flash Thinking Experimental (Google), and Llama 3.1 (Meta) [19–22]. Other popular models, including Grok and DeepSeek, were excluded due to token limitations and concerns over data privacy. Optional reasoning and search modules were enabled where applicable. Each LLM was accessed through publicly available web portals in February to April 2025.

### Prompt engineering

50 different prompts simulating hypothetical hemodialysis patients were created. Each prompt was designed to impose a range of nutritional, social and food preference constraints, varying in pre-specified domains, such as patient demographics, laboratory values, nutritional goals, and sociocultural factors to mirror the United States hemodialysis population.

National statistics from the United States Renal Data System [23] were used to create hypothetical patients with demographic variables that were representative of the US hemodialysis population. Age, gender, race and ethnicity were sampled from the prevalent US hemodialysis population, while BMI, weight, albumin, comorbidity, occupation, and insurance distribution were derived from incident hemodialysis patients [23]. Serum phosphorus data were simulated based off incident dialysis patient data from a large dialysis organization [24]. Food allergy, Celiac disease, and vegetarianism prevalence were derived from national surveys [25–27]. Food insecurity was simulated at 26.15% in accordance with recent estimates of food insecurity in the US hemodialysis population [28, 29]. Food preference was simulated for hypothetical patients with 50% of patients having no preference, with the remaining hypothetical patients equally favoring one of 34 randomly selected cuisines including Spanish, British, French, German, Italian, Greek, Saudi Arabian, Emirati, Indian, Chinese, Thai, Malaysian, Vietnamese, Korean, Filipino, Indonesian, Australian, Japanese, American, Mexican, and Caribbean cuisines. Food budget was estimated from normal distributions centered around $12 a day for individuals with a fixed income and $15 a day for individuals without a fixed income using suggested US Department of Agriculture food plans [30]. Fixed income status was ascribed to simulated patients who were retired, unemployed, or on disability using national occupational data [23].

With respect to patient-specific nutritional needs, pre-specified nutritional goals were created using National Kidney Foundation (NKF) guidelines [31], using standardized body weights [6, 32]. Calorie goals were simulated to be 35 kcal/kg/day for individuals < 60 years of age, and 30–35 kcal/kg/day for those 60 years and older, sampling from a normal distribution of mean 32.5 kcal/kg/day and standard deviation 1.25 kcal/kg/day. 33% of hypothetical patients were simulated to have protein energy wasting [33] and to have higher protein requirements of 1.3 g/kg/day compared to a standard 1.2 g/kg/day. Fiber goals were simulated from a normal distribution with mean 22.5 g/day and standard deviation 1.25 g/day. For criteria where NKF guidelines did not provide precise recommendations, expert opinion was used to set nutrition goals as follows: (1) Sodium – simulated from a log-normal distribution with µ = log(2000) and σ = 0.1, truncated to a minimum of 750 and max of 2000 mg/day, (2) Phosphorus – if serum phosphorus lab data was simulated to be > 5.5 mg/dL, the daily phosphorus maximum was sampled from a normal distribution of mean 12 mg/kg/day and standard deviation of 1 mg/kg/day, or a normal distribution of mean 14 mg/kg/day and standard deviation of 1 mg/kg/day if phosphorus was below 5.5 mg/dL, (3) Calcium – simulated from a log-normal distribution with µ = log(1000) and σ = 0.1, truncated to a minimum of 600 and max of 1000 mg/day, (4) Potassium - simulated from a log-normal distribution with µ = log(2730) and σ = 0.1, truncated to a minimum of 1800 and max of 3120 mg/day.

These criteria were synthesized into a conversational prompt and presented to each LLM without preamble using zero-shot prompting. LLMs were queried in separate sessions with privacy settings enabled when applicable. An example prompt is below:

I am a 62-year-old Asian man on hemodialysis three times a week. My dry weight is 87 kg and my BMI is 29.1. My dietitian recommends I eat a diet with 92 g of protein, 2150 calories, and 21 g of fiber per day. She also recommends no more than 850 mg of phosphorus, no more than 950 mg of calcium, no more than 1950 mg of sodium, and no more than 2900 mg per day of potassium. She also recommends drinking no more than 1100 mL of fluids per day. My albumin is 2.9 g/dL and my phosphorus is 6.4 mg/dL. I do not have Celiac disease. I have no allergies. I have no food preferences. My daily food budget is $12.5. I am experiencing food insecurity. I am not a vegetarian. I have heart failure. I have high blood pressure. I have diabetes. Can you help me with meal planning? Look it up and please provide a detailed meal plan that includes portion sizes. Please make sure to include a list of how much protein, calories, fiber, sodium, phosphorus, calcium, and potassium are in each meal, as well as the daily totals. Please only include a meal plan for a single day.

### Variables

**Fig. 1:**
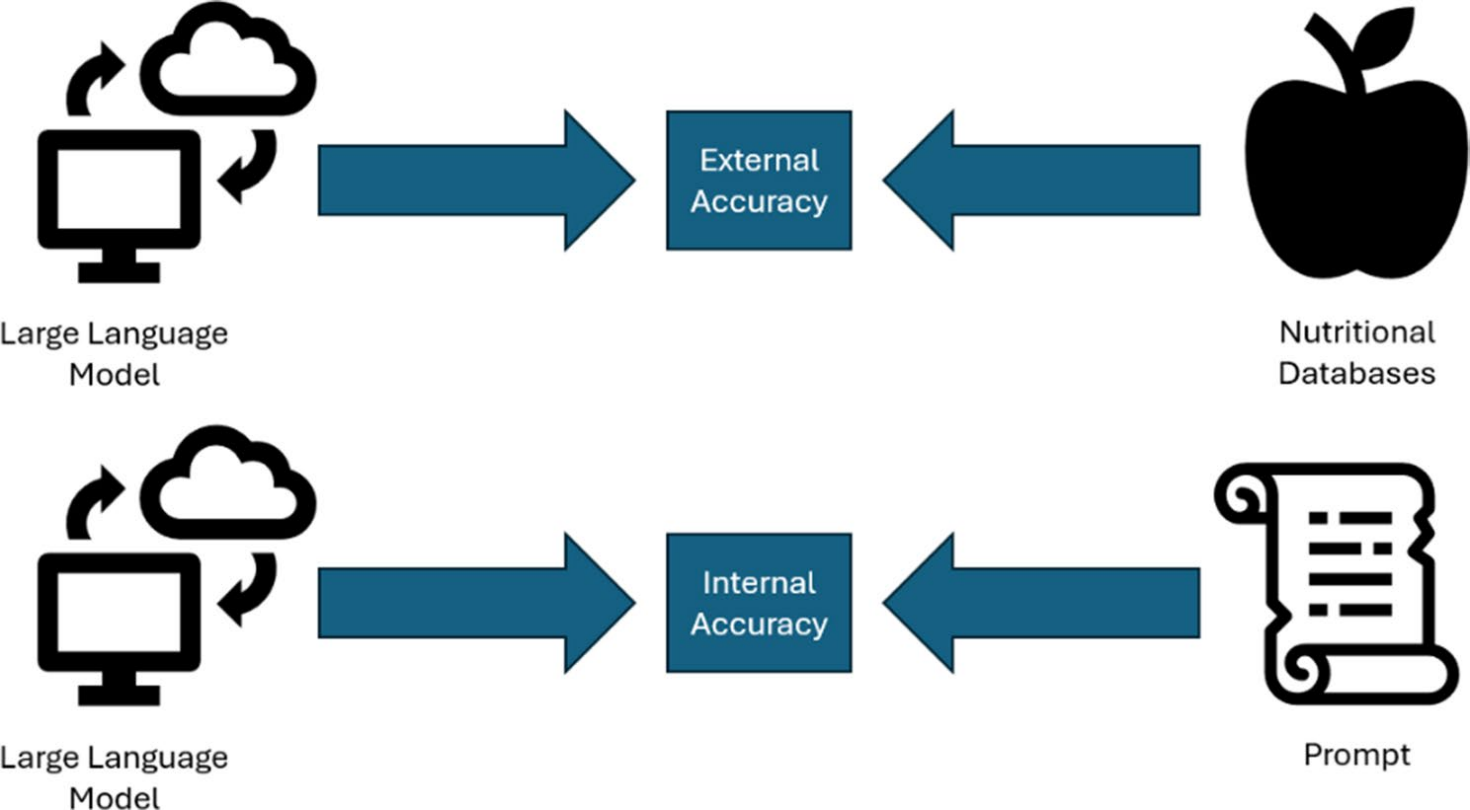
Schematic of external and internal accuracy

Our primary categorical predictor was the LLM used, either ChatGPT-o3-mini, Claude Sonnet 3.7, Gemini 2.5 Flash Thinking Experimental, or Llama 3.1. Our primary outcome was accuracy of nutritional recommendations, defined in two ways (Fig. 1). First, we defined external accuracy by comparing LLM output to a composite nutritional database made up of gold standard United States Department of Agriculture [34], Public Health England McCance and Widdowson [35], and Food Standards Australia New Zealand (AUSNUT) data. [36] Databases were accessed through a web-based utility called NutriAdmin [37], which was used to compile nutrition data. The accuracy of the NutriAdmin tool was independently verified against the source databases for an initial subset of LLM responses before using it for the broader analysis. Food items in LLMs were matched to database entries by name by three human reviewers (authors KS, HH, EC). The United States Department of Agriculture nutrient database was favored when foods were present in multiple sources. LLM outputs were considered externally accurate if stated nutritional contents were within 10% of the nutritional contents reported in the reference material. For example, if an LLM included a medium apple in its meal plan and reported that the apple had 25 calories, when reference nutritional database states a medium apple has 90 calories, the LLM would be considered inaccurate, as the LLM output would be beyond the 10% tolerance threshold from the reference material. External accuracy was assessed for daily meal plans cumulatively in the following domains: calories, protein, fiber, calcium, phosphorus, potassium, and sodium.

Second, internal accuracy was defined as the LLM outputs’ adherence to specific goals included in the prompt with nutritional outputs ± 10% away from the goal considered acceptable. For example, if a prompt asked for a meal plan with 2000 calories, AI-generated outputs with stated calories in the 1800–2200 calorie range would be considered accurate. Internal accuracy for the same domains of calories, protein, fiber, calcium, phosphorus, potassium, and sodium was calculated for each LLM. For our analysis, calories, protein, and fiber were considered macronutrients while calcium, phosphorus, potassium, and sodium were considered micronutrients.

To assess overall usability of LLM outputs, five types of qualitative errors were conceptualized: hallucinations, technical errors, atypical recommendations, vague instructions, and composite food errors. Hallucinations were defined as coherent but factually incorrect or illogical outputs. Technical errors were defined as improperly formatted answers, such as broken hyperlinks or nonsensical code snippets, or answers that clearly ignored prompt requirements, such as not outputting nutrient content for a recommended food. Atypical recommendations consisted of outputs that were not incorrect or illogical, but grossly out of normal nutritional practice, such as meeting a third of daily caloric needs with whole fats. Vague instructions were defined as outputs deemed to be too undetailed to be realistically helpful, such as outputting homemade food items but having no preparation instructions, such as “homemade muffin,” or describing a class of foods rather than a specific recommendation, such as “energy dense, low-potassium snacks.” Finally, composite food errors were defined as recommending composite food items without clear descriptions of components, such as “Chinese bun.” Presence or absence of qualitative errors were tabulated by three independent reviewers (KS, HH, EC), with the final subjective determination done by majority rule.

### Statistical analysis

Descriptive statistics were generated for the hypothetical patient cohort. External accuracy was calculated as the quotient of LLM output nutritional values that were within 10% of reference nutritional databases. Internal accuracy was calculated as the proportion of LLM output nutritional values that were within 10% of prompt goals. In the case of internal accuracy values, and prompt goals that were thresholds (i.e. do not exceed X), all responses that did not exceed 110% of the goal were considered accurate.

Differences between macronutrient (calories, protein, fiber) and micronutrient (calcium, phosphorus, potassium, sodium) accuracies were tested using paired Wilcoxon signed ranked tests. In this analysis, LLM results were split into paired groups of external and internal accuracy results for macronutrients and micronutrients, which were then compared. Results from separate LLM queries were treated as independent. Macronutrients were treated the same (e.g. calorie accuracy was not treated differently than protein accuracy) and micronutrients were treated the same (e.g. calcium accuracy was not treated differently than potassium accuracy).

For qualitative error analysis, a Wilcoxon ranked sum test was used to compare the cumulative qualitative errors amongst prompts with and without cuisine preferences. Results from separate LLM queries were treated as independent and types of errors (hallucinations, technical errors, etc.) were treated the same. All statistical analysis was done using R version 4.4 [38].

## Results

A sample of 50 hemodialysis patients was generated from nationally representative data (Table 1). The average age of simulated patients was 63 years (SD = 14), and the majority had hypertension (88%), diabetes (58%), and fixed incomes (82%).

**Table 1:** A sample of 50 simulated hemodialysis patient scenarios

Characteristic	Data
Total *N* = 50	
**Age in years (mean**,** (SD))**	63 (14)
**Sex (n**,** %)**	
Male	29 (58%)
Female	21 (42%)
**Race (n**,** %)**	
Asian	5 (10%)
Black	21 (42%)
Hispanic	8 (16%)
Native Hawaiian/Pacific Islander	1 (2%)
White	15 (30%)
**Biophysical factors (mean**,** (SD))**	
BMI (kg/m^2^)	29.7 (1.8)
Serum Albumin (mg/dL)	3.1 (0.3)
Serum Phosphorus (mg/dL)	5.0 (1.1)
**Comorbidities (n**,** %)**	
Hypertension	44 (88%)
Diabetes	29 (58%)
Congestive Heart Failure	14 (28%)
**Financial and Socioeconomic Factors (n**,** %)**	
Fixed income	41 (82%)
Medicaid	11 (22%)
Medicare	26 (52%)
Food Insecurity	10 (20%)
Food Budget in dollars per day (mean, (SD))	$12.60 (1.64)
**Food constraints (n**,** %)**	
Specific cuisine preference	29 (58%)
Vegetarian	1 (2%)
Fish allergy	1 (2%)
Peanut allergy	1 (2%)
**Simulated Nutrition Goals (mean**,** (SD))**	
Calories (kcal/day)	2430 (240)
Protein (g/day)	95 (8.4)
Fiber (g/day)	23 (1.3)
Calcium (mg/day)	< 952 (53)
Phosphorus (mg/day)	< 974 (125)
Potassium (mg/day)	< 2649 (227)
Sodium (mg/day)	< 1932 (97)
Fluid (mL/day)	< 1172 (99)

### Quantitative analysis

**Fig. 2:**
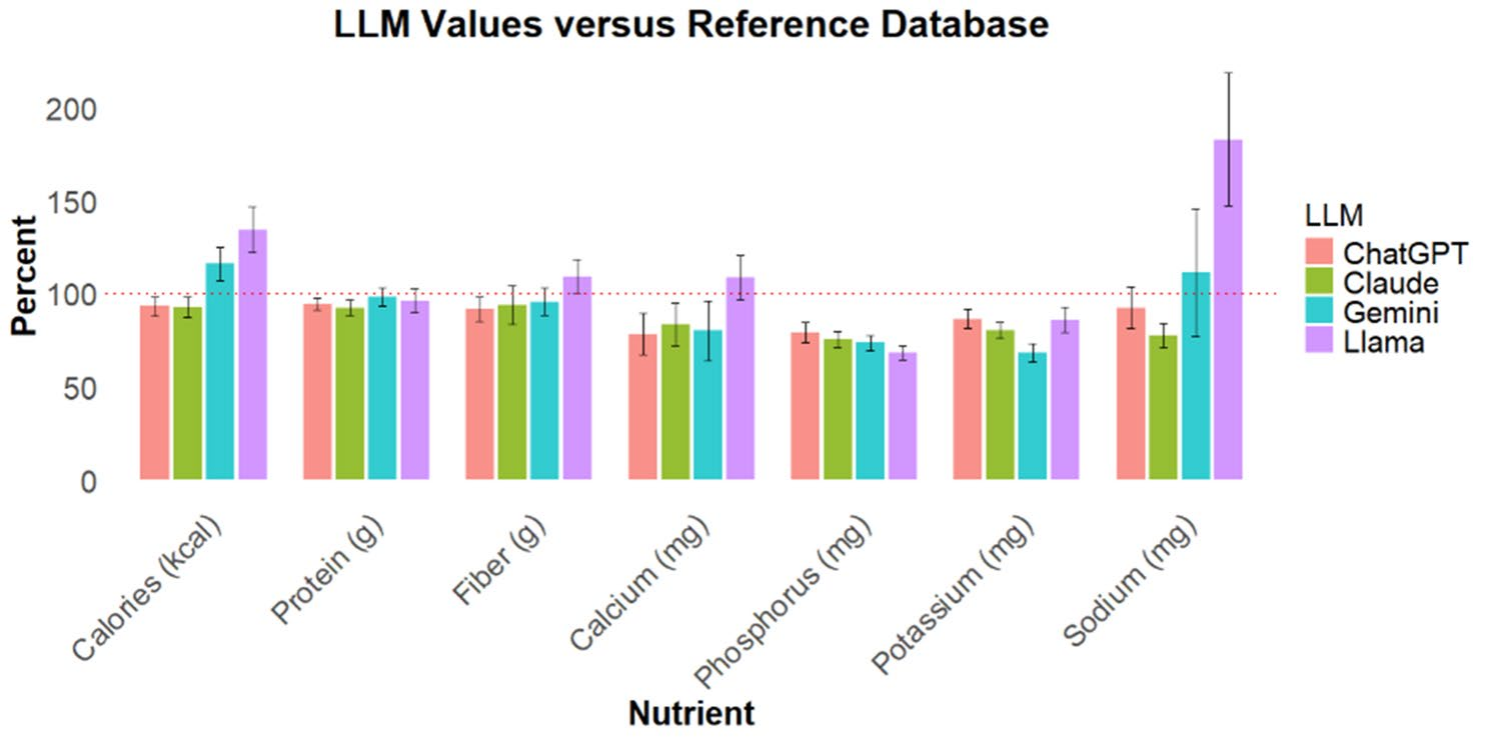
LLM estimates of nutritional content in meal plans as a percentage of reference values from established databases. Error bars represent the 95% confidence interval. The dotted line represents the gold standard reference value

Prompts describing each of these 50 patients and their nutritional goals were presented to ChatGPT, Claude, Gemini, and Llama LLMs. The stated nutritional content of chatbot meal plans was generally inaccurate when compared to gold standard values from governmental databases (Table 2). Next, though patterns of overestimation and underestimation varied across LLMs and nutrients, all underestimated phosphorus and potassium content in meal plans compared to gold standard databases (Fig. 2). In addition, external accuracy was worse for micronutrients, calcium, phosphorus, potassium, and sodium, compared to the macronutrients of calories, protein, or fiber (36.8% vs. 20.9% average discrepancy from gold standard; *p* < 0.001). Finally, despite being prompted, LLMs occasionally omitted presenting values for micronutrients (0–5 missing values per micronutrient per LLM), though calories, protein, and fiber were always included.

**Table 2:** –Percent of externally accurate LLM responses. LLM meal plans were considered accurate if reported results were within a 10% margin of reference database values

	External Accuracy						
LLM	Calories	Protein	Fiber	Calcium	Phosphorus	Potassium	Sodium
ChatGPT	42%	52%	28%	9%	24%	40%	18%
Claude	38%	34%	24%	8%	12%	10%	16%
Gemini	28%	50%	32%	10%	12%	8%	24%
Llama	12%	28%	26%	36%	6%	20%	8%

**Fig. 3:**
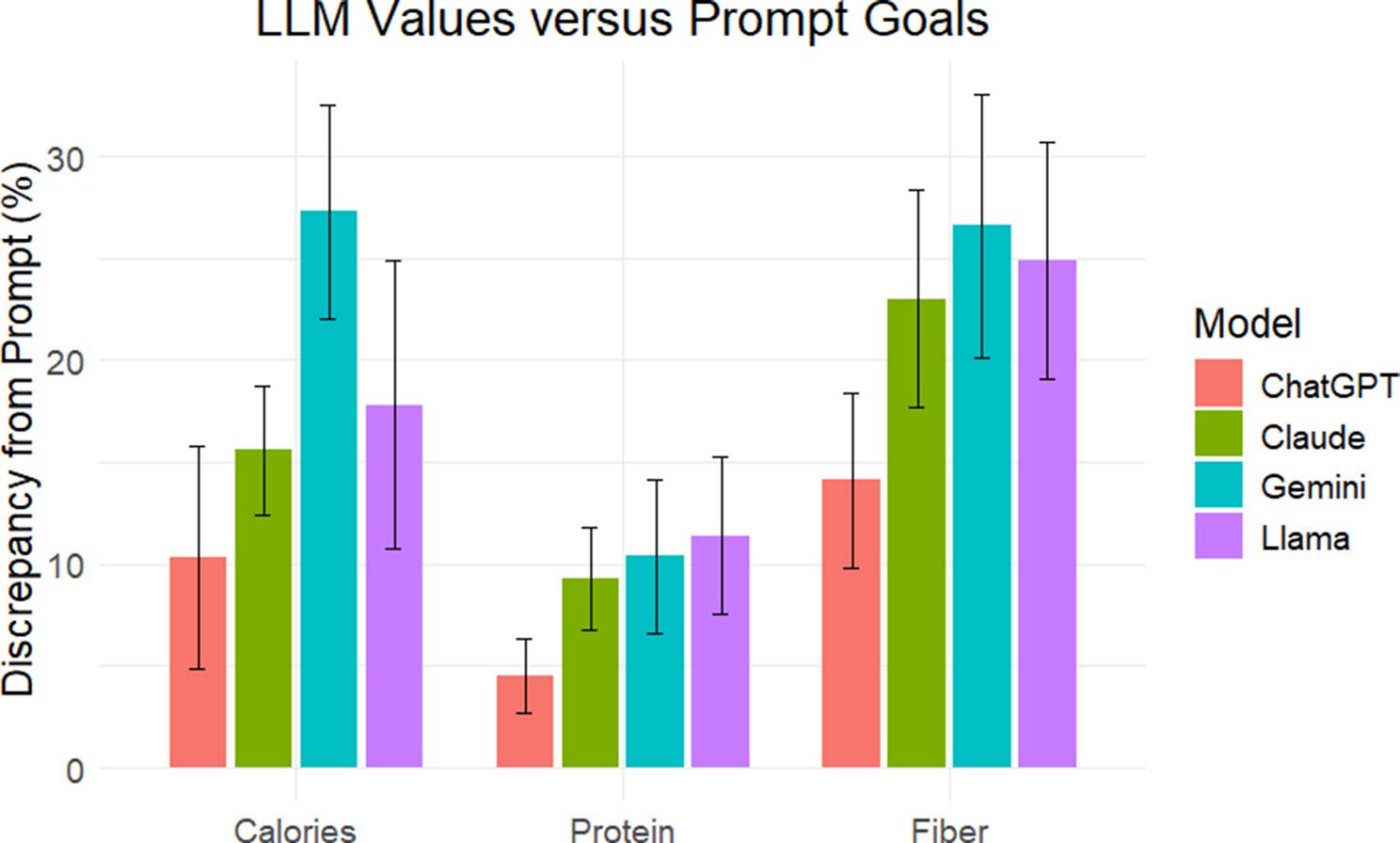
Internal accuracy for calories, protein, and fiber. Bars represent the discrepancy between nutritional content as presented by LLM outputs versus nutritional content demanded by prompts. Error bars represent the 95% confidence interval

When stated LLM outputs were compared to individualized nutrient goals stated in the prompt, internal accuracy was higher for micronutrients than for macronutrients (94.5% vs. 52.3% responses accurate; *p* < 0.001). Internal accuracy was high (> 90%) for micronutrients with all LLMs except Llama generally reporting appropriate values with respect to the prompt (Table 3). Using a ± 10% discrepancy threshold, internal accuracy of LLM output ranged from 24% to 86% for macronutrient goals. On average, ChatGPT outputs had the smallest percent deviation from prompt goals (Fig. 3).

**Table 3:** Percent of internally accurate LLM responses. LLM meal plans were considered accurate if reported results were within a 10% margin of prompt goals

	Internal Accuracy						
LLM	Calories	Protein	Fiber	Calcium	Phosphorus	Potassium	Sodium
ChatGPT	80%	86%	50%	98%	92%	96%	98%
Claude	30%	56%	38%	98%	92%	100%	100%
Gemini	24%	62%	34%	100%	96%	100%	100%
Llama	70%	70%	28%	98%	70%	76%	100%

### Qualitative analysis

Each LLM output was evaluated for the presence of 5 different types of qualitative errors by three independent reviewers. Qualitative errors that limited usability were common with all LLM outputs (Table 4). The most common type of error was in incompletely describing composite foods. Across all LLMs, errors were also relatively more common for prompts involving patients with an explicit cuisine preference (e.g. Thai cuisine, French cuisine) compared to those without a preference (1 error per response vs. 0.48 errors per response; *p* < 0.001).

**Table 4:** Percentage of LLM outputs with qualitative errors

Qualitative Error Type	ChatGPT	Claude	Gemini	Llama	Example(s)
Hallucinations	0%	0%	0%	0%	None
Technical Error	6%	0%	4%	0%	Inclusion of code snippet: *tool_code print(Google Search(queries=[“*
Atypical Recommendations	34%	4%	2%	0%	Drinking olive oil “shots”
Vague Instructions	30%	38%	22%	8%	Homemade Shepard’s Pie (no instructions on preparation)
Composite Food Error	52%	70%	20%	22%	Spanish Flan (no ingredients listed)

## Discussion

With this study, we demonstrate that, from February to April 2025, four highly ranked general LLMs were unable to provide accurate and feasible meal plans for hypothetical patients with ESKD requiring hemodialysis with personalized nutritional goals.

External accuracy, a metric designed to evaluate LLMs’ abilities to extract or recall information from trusted sources, was generally low across LLMs. Notably, all LLMs underestimated the true potassium and phosphorus contents in their proposed meal plans, which could confer a risk of hyperkalemia and hyperphosphatemia in clinical practice. In some cases, LLMs may fail to consistently find, recognize, or prefer trusted sources over less validated references. For this reason, techniques to enhance the knowledge base of LLMs, such as retrieval-augmented generation, may improve performance. For example, Gençer Bingöl et al. found that incorporating the Kidney Disease Outcomes Quality Initiative Clinical Practice Guidelines into the knowledge context of Llama 2.7b improved the accuracy of nutritional advice. [39] Beyond this, it is also important to recognize deficiencies in dietary resources. For example, phosphorus remains a non-mandatory component of US nutrition labels [40], limiting the potential accuracy of any model, even if its ability to search and extract high quality data is exceptional. Similarly, the US Department of Agriculture has detailed, high-quality nutritional information on staple Western foods, but does not include many ethnic foods from other cultures. Enhancing model performance in these cases requires basic nutritional science rather than changing software parameters or context.

Internal accuracy, a metric designed to assess LLMs’ abilities to properly manipulate presented quantitative data, was also low across models. Although models often succeeded when the specified requirement was to remain below a threshold (e.g., sodium or phosphorus limits), they frequently failed when asked to achieve specific quantitative goals, as was requested for the macronutrients of calories, protein, and fiber. In most cases, generated plans undershot goals, reflecting either a difficulty with numeric reasoning or an inappropriate tolerance for error. This may stem from the underlying probabilistic methods that LLMs use for content generation, and utilization of deterministic, rather than probabilistic, techniques may enhance performance. For example, instead of predicting the next word in a sequence to generate output, a hybrid approach that leverages computational methods for constrained optimization and complex mathematical tasks but retains the flexible conversational format LLMs for input and output may better align with strict numeric goals.

When the quality of LLM outputs was judged by human readers, errors that limited usability were common. Future usability research from direct stakeholders, such as patients and dietitians, will be critical. Critically, more errors were present in answers to prompts that asked for specific ethnic cuisines. This discrepancy raises health equity concerns and may limit the utility of LLMs for meal planning among patients with ESKD, given the disproportionate burden of kidney disease among minoritized populations who also consume more diverse foods. [23].

While the studied LLMs are convenient and may be useful for preliminary explorations of dietary options, these deficiencies highlight the need for end user awareness and human oversight in meal planning. Currently, these tools are best used as an adjunct to collaborative patient-provider discussions with renal dietitians, rather than independent or authoritative resources for precision nutrition.

This study makes several contributions to the growing body of literature on this topic. It extends prior evaluations of LLMs into the ESKD domain, where dietary constraints are particularly complex and clinically consequential. We simulated diverse patient scenarios incorporating not only biometric and comorbidity data but also social factors such as dietary preferences and food budgets, approximating the true clinical diversity faced by kidney providers. The study also used a broad but verified knowledge base of three federal nutritional databases as its basis for accuracy. Finally, we combined quantitative and qualitative approaches for a comprehensive evaluation.

This study also has several limitations. First, we used a 10% deviation threshold to classify outputs as accurate or inaccurate. While this provided a consistent analytic framework, its clinical significance is unclear. Clinical consequences of diets in the ESKD context depend on a multitude of patient factors including residual renal function, comorbidities, and nutrient metabolism, which could not be assessed in this hypothetical patient scenario. Moreover, LLMs inconsistently reported certain critical parameters, such as fluid intake, even when explicitly prompted. For this reason, analysis of fluid intake was excluded despite its central role in dialysis nutrition. Similarly, cost estimates and budget considerations could not be evaluated because models often omitted or vaguely reported pricing. Second, LLM technology evolves rapidly and our results may not apply to future model versions, though our results are globally similar to past evaluations of previous models, suggesting that undirected global improvements may not meaningfully address LLM deficiencies in this space. [15, 17] Third, qualitative evaluations were conducted by non-dietitians (authors KS, HH, and EC), which limits generalizability to MNT settings. Finally, to enable direct comparisons across LLMs, singular prompts, without follow-up or clarification, were used in this study; continued contextual engagement with LLMs could improve performance.

Ultimately, these results suggest that existing general purpose LLMs, assessed from February to April 2025, cannot yet be relied upon to provide accurate support for renal dietitians. Patients should be informed of these tools’ limited use as independent resources. Significant improvements are necessary before these tools can be clinically applied in the highly restrictive context of nutrition in ESKD. Creation of a truly useful precision nutrition counseling LLM will require domain-specific model enhancement and customization, basic research to generate more diverse and comprehensive nutritional data, and stakeholder-led qualitative evaluations of usability and clarity. This will require active investment and future research and collaboration from nephrologists, dietitians, data and food scientists, health systems leaders, and patients.

## Conclusion

Despite rapid improvement in the past three years, current large language models have significant quantitative and qualitative deficits that should preclude their use in meal planning for individuals with end stage kidney disease. While these tools have immense potential to improve dietary counseling workflows, this potential has yet to be realized. Patients and providers should currently use these tools with caution, especially as large language models become more prevalent in daily life. Further research in not only software development, but food and implementation science will be necessary to drive progress.

## Supplementary Information

Below is the link to the electronic supplementary material.

Supplementary Material 1

Supplementary Material 2

## Data Availability

The datasets and code used and/or analyzed during the current study are available from the corresponding author on reasonable request.

## Abbreviations

ESKD: End stage kidney disease
MNT: Medical nutrition therapy
LLM: Large language model
