# Supplementary material for: Assessment of large language model chatbots for hemodialysis meal planning: a descriptive study
Source: BMC Nephrol. 2026 Mar 31;27:298. doi: 10.1186/s12882-026-04936-8 (PMC13162381; doi:10.1186/s12882-026-04936-8)
Supplement: Supplementary file 1 — Supplementary Material 1 [file 12882_2026_4936_MOESM1_ESM.docx]

Supplement S1 – List of Prompts for 50 Simulated Hemodialysis Patients. Prompts are identical except for select parameters (age, sex, weight, nutrition goals, etc.).

| 1 | I am a 53-year-old White man on hemodialysis three times a week. My dry weight is 94 kg and my BMI is 29.6. My dietitian recommends I eat a diet with 116 g of protein, 3100 calories, and 19 g of fiber per day. She also recommends no more than 1250 mg of phosphorus, no more than 900 mg of calcium, no more than 1900 mg of sodium, and no more than 2650 mg per day of potassium. She also recommends drinking no more than 1200 mL of fluids per day. My albumin is 3g/dL and my phosphorus is 4.7 mg/dL. I do not have Celiac disease. I have no allergies. My favorite cuisine is French cuisine. My daily food budget is $12.5. I am not experiencing food insecurity. I am not a vegetarian. I have heart failure. I have diabetes. Can you help me with meal planning? Look it up and please provide a detailed meal plan that includes portion sizes. Please make sure to include a list of how much protein, calories, fiber, sodium, phosphorus, calcium, and potassium are in each meal, as well as the daily totals. Please only include a meal plan for a single day. |
| --- | --- |
| 2 | I am a 44-year-old White woman on hemodialysis three times a week. My dry weight is 92 kg and my BMI is 32.3. My dietitian recommends I eat a diet with 85 g of protein, 2300 calories, and 22 g of fiber per day. She also recommends no more than 950 mg of phosphorus, no more than 900 mg of calcium, no more than 1900 mg of sodium, and no more than 3000 mg per day of potassium. She also recommends drinking no more than 1300 mL of fluids per day. My albumin is 3.3g/dL and my phosphorus is 3.6 mg/dL. I do not have Celiac disease. I have no allergies. My favorite cuisine is Lebanese cuisine. My daily food budget is $11.5. I am not experiencing food insecurity. I am not a vegetarian. I have high blood pressure. I have diabetes. Can you help me with meal planning? Look it up and please provide a detailed meal plan that includes portion sizes. Please make sure to include a list of how much protein, calories, fiber, sodium, phosphorus, calcium, and potassium are in each meal, as well as the daily totals. Please only include a meal plan for a single day. |
| 3 | I am a 62-year-old Black woman on hemodialysis three times a week. My dry weight is 83 kg and my BMI is 30.4. My dietitian recommends I eat a diet with 88 g of protein, 2150 calories, and 22 g of fiber per day. She also recommends no more than 900 mg of phosphorus, no more than 1000 mg of calcium, no more than 2000 mg of sodium, and no more than 2900 mg per day of potassium. She also recommends drinking no more than 1100 mL of fluids per day. My albumin is 3.2g/dL and my phosphorus is 3.8 mg/dL. I do not have Celiac disease. I have no allergies. My favorite cuisine is Argentinian cuisine. My daily food budget is $12. I am not experiencing food insecurity. I am not a vegetarian. I have diabetes. Can you help me with meal planning? Look it up and please provide a detailed meal plan that includes portion sizes. Please make sure to include a list of how much protein, calories, fiber, sodium, phosphorus, calcium, and potassium are in each meal, as well as the daily totals. Please only include a meal plan for a single day. |
| 4 | I am a 62-year-old Asian man on hemodialysis three times a week. My dry weight is 87 kg and my BMI is 29.1. My dietitian recommends I eat a diet with 92 g of protein, 2150 calories, and 21 g of fiber per day. She also recommends no more than 850 mg of phosphorus, no more than 950 mg of calcium, no more than 1950 mg of sodium, and no more than 2900 mg per day of potassium. She also recommends drinking no more than 1100 mL of fluids per day. My albumin is 2.9g/dL and my phosphorus is 6.4 mg/dL. I do not have Celiac disease. I have no allergies. I have no food preferences. My daily food budget is $12.5. I am experiencing food insecurity. I am not a vegetarian. I have heart failure. I have high blood pressure. I have diabetes. Can you help me with meal planning? Look it up and please provide a detailed meal plan that includes portion sizes. Please make sure to include a list of how much protein, calories, fiber, sodium, phosphorus, calcium, and potassium are in each meal, as well as the daily totals. Please only include a meal plan for a single day. |
| 5 | I am a 58-year-old Asian woman on hemodialysis three times a week. My dry weight is 89 kg and my BMI is 32.1. My dietitian recommends I eat a diet with 88 g of protein, 2400 calories, and 23 g of fiber per day. She also recommends no more than 1000 mg of phosphorus, no more than 950 mg of calcium, no more than 1850 mg of sodium, and no more than 2500 mg per day of potassium. She also recommends drinking no more than 1300 mL of fluids per day. My albumin is 3.2g/dL and my phosphorus is 4.4 mg/dL. I do not have Celiac disease. I have no allergies. My favorite cuisine is Thai cuisine. My daily food budget is $12. I am not experiencing food insecurity. I am not a vegetarian. I have high blood pressure. I have diabetes. Can you help me with meal planning? Look it up and please provide a detailed meal plan that includes portion sizes. Please make sure to include a list of how much protein, calories, fiber, sodium, phosphorus, calcium, and potassium are in each meal, as well as the daily totals. Please only include a meal plan for a single day. |
| 6 | I am a 73-year-old White man on hemodialysis three times a week. My dry weight is 83 kg and my BMI is 29.6. My dietitian recommends I eat a diet with 96 g of protein, 2400 calories, and 24 g of fiber per day. She also recommends no more than 750 mg of phosphorus, no more than 900 mg of calcium, no more than 2000 mg of sodium, and no more than 2250 mg per day of potassium. She also recommends drinking no more than 1200 mL of fluids per day. My albumin is 3.6g/dL and my phosphorus is 7.2 mg/dL. I do not have Celiac disease. I have no allergies. My favorite cuisine is Emirati cuisine. My daily food budget is $10.5. I am not experiencing food insecurity. I am not a vegetarian. I have high blood pressure. Can you help me with meal planning? Look it up and please provide a detailed meal plan that includes portion sizes. Please make sure to include a list of how much protein, calories, fiber, sodium, phosphorus, calcium, and potassium are in each meal, as well as the daily totals. Please only include a meal plan for a single day. |
| 7 | I am a 59-year-old Black woman on hemodialysis three times a week. My dry weight is 90 kg and my BMI is 30.5. My dietitian recommends I eat a diet with 91 g of protein, 2450 calories, and 25 g of fiber per day. She also recommends no more than 800 mg of phosphorus, no more than 1000 mg of calcium, no more than 2000 mg of sodium, and no more than 2350 mg per day of potassium. She also recommends drinking no more than 1000 mL of fluids per day. My albumin is 3.3g/dL and my phosphorus is 5.7 mg/dL. I do not have Celiac disease. I have no allergies. My favorite cuisine is Chinese cuisine. My daily food budget is $12. I am not experiencing food insecurity. I am not a vegetarian. I have high blood pressure. Can you help me with meal planning? Look it up and please provide a detailed meal plan that includes portion sizes. Please make sure to include a list of how much protein, calories, fiber, sodium, phosphorus, calcium, and potassium are in each meal, as well as the daily totals. Please only include a meal plan for a single day. |
| 8 | I am a 35-year-old Hispanic woman on hemodialysis three times a week. My dry weight is 90 kg and my BMI is 31.9. My dietitian recommends I eat a diet with 85 g of protein, 2300 calories, and 19 g of fiber per day. She also recommends no more than 850 mg of phosphorus, no more than 950 mg of calcium, no more than 2000 mg of sodium, and no more than 2750 mg per day of potassium. She also recommends drinking no more than 1100 mL of fluids per day. My albumin is 3.2g/dL and my phosphorus is 5.4 mg/dL. I do not have Celiac disease. I have no allergies. I have no food preferences. My daily food budget is $12. I am experiencing food insecurity. I am not a vegetarian. I have high blood pressure. Can you help me with meal planning? Look it up and please provide a detailed meal plan that includes portion sizes. Please make sure to include a list of how much protein, calories, fiber, sodium, phosphorus, calcium, and potassium are in each meal, as well as the daily totals. Please only include a meal plan for a single day. |
| 9 | I am a 52-year-old White woman on hemodialysis three times a week. My dry weight is 93 kg and my BMI is 32.2. My dietitian recommends I eat a diet with 85 g of protein, 2300 calories, and 23 g of fiber per day. She also recommends no more than 900 mg of phosphorus, no more than 950 mg of calcium, no more than 1900 mg of sodium, and no more than 2650 mg per day of potassium. She also recommends drinking no more than 1000 mL of fluids per day. My albumin is 3.1g/dL and my phosphorus is 6.8 mg/dL. I do not have Celiac disease. I have no allergies. I have no food preferences. My daily food budget is $17.5. I am not experiencing food insecurity. I am not a vegetarian. I have high blood pressure. I have diabetes. Can you help me with meal planning? Look it up and please provide a detailed meal plan that includes portion sizes. Please make sure to include a list of how much protein, calories, fiber, sodium, phosphorus, calcium, and potassium are in each meal, as well as the daily totals. Please only include a meal plan for a single day. |
| 10 | I am a 71-year-old Black woman on hemodialysis three times a week. My dry weight is 82 kg and my BMI is 30. My dietitian recommends I eat a diet with 88 g of protein, 2300 calories, and 21 g of fiber per day. She also recommends no more than 950 mg of phosphorus, no more than 900 mg of calcium, no more than 1900 mg of sodium, and no more than 2500 mg per day of potassium. She also recommends drinking no more than 1100 mL of fluids per day. My albumin is 2.6g/dL and my phosphorus is 3.1 mg/dL. I do not have Celiac disease. I have no allergies. My favorite cuisine is French cuisine. My daily food budget is $12.5. I am not experiencing food insecurity. I am not a vegetarian. I have high blood pressure. I have diabetes. Can you help me with meal planning? Look it up and please provide a detailed meal plan that includes portion sizes. Please make sure to include a list of how much protein, calories, fiber, sodium, phosphorus, calcium, and potassium are in each meal, as well as the daily totals. Please only include a meal plan for a single day. |
| 11 | I am a 91-year-old Asian man on hemodialysis three times a week. My dry weight is 72 kg and my BMI is 26.5. My dietitian recommends I eat a diet with 94 g of protein, 2300 calories, and 24 g of fiber per day. She also recommends no more than 900 mg of phosphorus, no more than 950 mg of calcium, no more than 2000 mg of sodium, and no more than 2600 mg per day of potassium. She also recommends drinking no more than 1400 mL of fluids per day. My albumin is 3.5g/dL and my phosphorus is 6.4 mg/dL. I do not have Celiac disease. I have no allergies. My favorite cuisine is Brazilian cuisine. My daily food budget is $12.5. I am not experiencing food insecurity. I am not a vegetarian. I have heart failure. I have high blood pressure. Can you help me with meal planning? Look it up and please provide a detailed meal plan that includes portion sizes. Please make sure to include a list of how much protein, calories, fiber, sodium, phosphorus, calcium, and potassium are in each meal, as well as the daily totals. Please only include a meal plan for a single day. |
| 12 | I am a 83-year-old White man on hemodialysis three times a week. My dry weight is 79 kg and my BMI is 27.7. My dietitian recommends I eat a diet with 98 g of protein, 2400 calories, and 23 g of fiber per day. She also recommends no more than 850 mg of phosphorus, no more than 950 mg of calcium, no more than 2000 mg of sodium, and no more than 2750 mg per day of potassium. She also recommends drinking no more than 1300 mL of fluids per day. My albumin is 2.8g/dL and my phosphorus is 7.1 mg/dL. I do not have Celiac disease. I am allergic to fish. I have no food preferences. My daily food budget is $13. I am not experiencing food insecurity. I am not a vegetarian. I have heart failure. I have high blood pressure. I have diabetes. Can you help me with meal planning? Look it up and please provide a detailed meal plan that includes portion sizes. Please make sure to include a list of how much protein, calories, fiber, sodium, phosphorus, calcium, and potassium are in each meal, as well as the daily totals. Please only include a meal plan for a single day. |
| 13 | I am a 70-year-old Hispanic man on hemodialysis three times a week. My dry weight is 84 kg and my BMI is 29.1. My dietitian recommends I eat a diet with 99 g of protein, 2450 calories, and 24 g of fiber per day. She also recommends no more than 1000 mg of phosphorus, no more than 900 mg of calcium, no more than 2000 mg of sodium, and no more than 2650 mg per day of potassium. She also recommends drinking no more than 1200 mL of fluids per day. My albumin is 3.4g/dL and my phosphorus is 4 mg/dL. I do not have Celiac disease. I have no allergies. I have no food preferences. My daily food budget is $11.5. I am not experiencing food insecurity. I am not a vegetarian. I have high blood pressure. I have diabetes. Can you help me with meal planning? Look it up and please provide a detailed meal plan that includes portion sizes. Please make sure to include a list of how much protein, calories, fiber, sodium, phosphorus, calcium, and potassium are in each meal, as well as the daily totals. Please only include a meal plan for a single day. |
| 14 | I am a 68-year-old Hispanic man on hemodialysis three times a week. My dry weight is 84 kg and my BMI is 29.5. My dietitian recommends I eat a diet with 106 g of protein, 2550 calories, and 21 g of fiber per day. She also recommends no more than 1150 mg of phosphorus, no more than 950 mg of calcium, no more than 1800 mg of sodium, and no more than 2600 mg per day of potassium. She also recommends drinking no more than 1300 mL of fluids per day. My albumin is 3.8g/dL and my phosphorus is 4.8 mg/dL. I do not have Celiac disease. I have no allergies. I have no food preferences. My daily food budget is $11.5. I am not experiencing food insecurity. I am not a vegetarian. I have high blood pressure. I have diabetes. Can you help me with meal planning? Look it up and please provide a detailed meal plan that includes portion sizes. Please make sure to include a list of how much protein, calories, fiber, sodium, phosphorus, calcium, and potassium are in each meal, as well as the daily totals. Please only include a meal plan for a single day. |
| 15 | I am a 61-year-old White man on hemodialysis three times a week. My dry weight is 90 kg and my BMI is 29.8. My dietitian recommends I eat a diet with 103 g of protein, 2550 calories, and 23 g of fiber per day. She also recommends no more than 850 mg of phosphorus, no more than 1000 mg of calcium, no more than 2000 mg of sodium, and no more than 2550 mg per day of potassium. She also recommends drinking no more than 1200 mL of fluids per day. My albumin is 2.8g/dL and my phosphorus is 5.6 mg/dL. I do not have Celiac disease. I have no allergies. I have no food preferences. My daily food budget is $11. I am not experiencing food insecurity. I am not a vegetarian. I have high blood pressure. I have diabetes. Can you help me with meal planning? Look it up and please provide a detailed meal plan that includes portion sizes. Please make sure to include a list of how much protein, calories, fiber, sodium, phosphorus, calcium, and potassium are in each meal, as well as the daily totals. Please only include a meal plan for a single day. |
| 16 | I am a 80-year-old Hispanic woman on hemodialysis three times a week. My dry weight is 79 kg and my BMI is 26.4. My dietitian recommends I eat a diet with 92 g of protein, 2350 calories, and 23 g of fiber per day. She also recommends no more than 900 mg of phosphorus, no more than 950 mg of calcium, no more than 2000 mg of sodium, and no more than 2750 mg per day of potassium. She also recommends drinking no more than 1300 mL of fluids per day. My albumin is 3.4g/dL and my phosphorus is 3.4 mg/dL. I do not have Celiac disease. I have no allergies. I have no food preferences. My daily food budget is $13. I am experiencing food insecurity. I am a vegetarian. I have high blood pressure. I have diabetes. Can you help me with meal planning? Look it up and please provide a detailed meal plan that includes portion sizes. Please make sure to include a list of how much protein, calories, fiber, sodium, phosphorus, calcium, and potassium are in each meal, as well as the daily totals. Please only include a meal plan for a single day. |
| 17 | I am a 64-year-old White woman on hemodialysis three times a week. My dry weight is 88 kg and my BMI is 30. My dietitian recommends I eat a diet with 91 g of protein, 2350 calories, and 24 g of fiber per day. She also recommends no more than 1150 mg of phosphorus, no more than 1000 mg of calcium, no more than 1850 mg of sodium, and no more than 2900 mg per day of potassium. She also recommends drinking no more than 1200 mL of fluids per day. My albumin is 3.1g/dL and my phosphorus is 5.3 mg/dL. I do not have Celiac disease. I have no allergies. I have no food preferences. My daily food budget is $12.5. I am not experiencing food insecurity. I am not a vegetarian. I have heart failure. I have diabetes. Can you help me with meal planning? Look it up and please provide a detailed meal plan that includes portion sizes. Please make sure to include a list of how much protein, calories, fiber, sodium, phosphorus, calcium, and potassium are in each meal, as well as the daily totals. Please only include a meal plan for a single day. |
| 18 | I am a 71-year-old White man on hemodialysis three times a week. My dry weight is 83 kg and my BMI is 27.9. My dietitian recommends I eat a diet with 102 g of protein, 2650 calories, and 22 g of fiber per day. She also recommends no more than 1150 mg of phosphorus, no more than 1000 mg of calcium, no more than 2000 mg of sodium, and no more than 2350 mg per day of potassium. She also recommends drinking no more than 1100 mL of fluids per day. My albumin is 2.7g/dL and my phosphorus is 4.4 mg/dL. I do not have Celiac disease. I have no allergies. My favorite cuisine is British cuisine. My daily food budget is $11. I am experiencing food insecurity. I am not a vegetarian. I have heart failure. I have high blood pressure. Can you help me with meal planning? Look it up and please provide a detailed meal plan that includes portion sizes. Please make sure to include a list of how much protein, calories, fiber, sodium, phosphorus, calcium, and potassium are in each meal, as well as the daily totals. Please only include a meal plan for a single day. |
| 19 | I am a 68-year-old Black man on hemodialysis three times a week. My dry weight is 83 kg and my BMI is 32. My dietitian recommends I eat a diet with 83 g of protein, 2050 calories, and 24 g of fiber per day. She also recommends no more than 900 mg of phosphorus, no more than 1000 mg of calcium, no more than 1950 mg of sodium, and no more than 2950 mg per day of potassium. She also recommends drinking no more than 1200 mL of fluids per day. My albumin is 2.7g/dL and my phosphorus is 5.5 mg/dL. I do not have Celiac disease. I have no allergies. I have no food preferences. My daily food budget is $13. I am not experiencing food insecurity. I am not a vegetarian. I have high blood pressure. I have diabetes. Can you help me with meal planning? Look it up and please provide a detailed meal plan that includes portion sizes. Please make sure to include a list of how much protein, calories, fiber, sodium, phosphorus, calcium, and potassium are in each meal, as well as the daily totals. Please only include a meal plan for a single day. |
| 20 | I am a 81-year-old Asian woman on hemodialysis three times a week. My dry weight is 80 kg and my BMI is 28.3. My dietitian recommends I eat a diet with 89 g of protein, 2200 calories, and 22 g of fiber per day. She also recommends no more than 900 mg of phosphorus, no more than 900 mg of calcium, no more than 1800 mg of sodium, and no more than 2550 mg per day of potassium. She also recommends drinking no more than 1100 mL of fluids per day. My albumin is 2.7g/dL and my phosphorus is 4.8 mg/dL. I do not have Celiac disease. I have no allergies. My favorite cuisine is German cuisine. My daily food budget is $11. I am experiencing food insecurity. I am not a vegetarian. I have high blood pressure. Can you help me with meal planning? Look it up and please provide a detailed meal plan that includes portion sizes. Please make sure to include a list of how much protein, calories, fiber, sodium, phosphorus, calcium, and potassium are in each meal, as well as the daily totals. Please only include a meal plan for a single day. |
| 21 | I am a 80-year-old Hispanic man on hemodialysis three times a week. My dry weight is 74 kg and my BMI is 26. My dietitian recommends I eat a diet with 98 g of protein, 2500 calories, and 22 g of fiber per day. She also recommends no more than 950 mg of phosphorus, no more than 1000 mg of calcium, no more than 1600 mg of sodium, and no more than 3000 mg per day of potassium. She also recommends drinking no more than 1200 mL of fluids per day. My albumin is 3g/dL and my phosphorus is 5.8 mg/dL. I do not have Celiac disease. I have no allergies. My favorite cuisine is Spanish cuisine. My daily food budget is $12.5. I am not experiencing food insecurity. I am not a vegetarian. I have high blood pressure. I have diabetes. Can you help me with meal planning? Look it up and please provide a detailed meal plan that includes portion sizes. Please make sure to include a list of how much protein, calories, fiber, sodium, phosphorus, calcium, and potassium are in each meal, as well as the daily totals. Please only include a meal plan for a single day. |
| 22 | I am a 47-year-old Black woman on hemodialysis three times a week. My dry weight is 99 kg and my BMI is 31.2. My dietitian recommends I eat a diet with 99 g of protein, 2650 calories, and 23 g of fiber per day. She also recommends no more than 1000 mg of phosphorus, no more than 1000 mg of calcium, no more than 2000 mg of sodium, and no more than 2400 mg per day of potassium. She also recommends drinking no more than 1000 mL of fluids per day. My albumin is 2.7g/dL and my phosphorus is 4.9 mg/dL. I do not have Celiac disease. I have no allergies. I have no food preferences. My daily food budget is $13.5. I am not experiencing food insecurity. I am not a vegetarian. I have high blood pressure. Can you help me with meal planning? Look it up and please provide a detailed meal plan that includes portion sizes. Please make sure to include a list of how much protein, calories, fiber, sodium, phosphorus, calcium, and potassium are in each meal, as well as the daily totals. Please only include a meal plan for a single day. |
| 23 | I am a 75-year-old Black woman on hemodialysis three times a week. My dry weight is 84 kg and my BMI is 27.8. My dietitian recommends I eat a diet with 106 g of protein, 2750 calories, and 21 g of fiber per day. She also recommends no more than 1200 mg of phosphorus, no more than 1000 mg of calcium, no more than 2000 mg of sodium, and no more than 2800 mg per day of potassium. She also recommends drinking no more than 1200 mL of fluids per day. My albumin is 3.4g/dL and my phosphorus is 4.5 mg/dL. I do not have Celiac disease. I have no allergies. My favorite cuisine is Japanese cuisine. My daily food budget is $12. I am experiencing food insecurity. I am not a vegetarian. I have high blood pressure. I have diabetes. Can you help me with meal planning? Look it up and please provide a detailed meal plan that includes portion sizes. Please make sure to include a list of how much protein, calories, fiber, sodium, phosphorus, calcium, and potassium are in each meal, as well as the daily totals. Please only include a meal plan for a single day. |
| 24 | I am a 41-year-old Native Hawaiian/Pacific Islander man on hemodialysis three times a week. My dry weight is 92 kg and my BMI is 32.3. My dietitian recommends I eat a diet with 89 g of protein, 2400 calories, and 22 g of fiber per day. She also recommends no more than 1100 mg of phosphorus, no more than 1000 mg of calcium, no more than 2000 mg of sodium, and no more than 2750 mg per day of potassium. She also recommends drinking no more than 1200 mL of fluids per day. My albumin is 3.8g/dL and my phosphorus is 5.5 mg/dL. I do not have Celiac disease. I have no allergies. My favorite cuisine is Indian cuisine. My daily food budget is $13. I am not experiencing food insecurity. I am not a vegetarian. I have high blood pressure. Can you help me with meal planning? Look it up and please provide a detailed meal plan that includes portion sizes. Please make sure to include a list of how much protein, calories, fiber, sodium, phosphorus, calcium, and potassium are in each meal, as well as the daily totals. Please only include a meal plan for a single day. |
| 25 | I am a 42-year-old Black man on hemodialysis three times a week. My dry weight is 90 kg and my BMI is 31.4. My dietitian recommends I eat a diet with 98 g of protein, 2650 calories, and 24 g of fiber per day. She also recommends no more than 900 mg of phosphorus, no more than 1000 mg of calcium, no more than 1900 mg of sodium, and no more than 2200 mg per day of potassium. She also recommends drinking no more than 1200 mL of fluids per day. My albumin is 2.8g/dL and my phosphorus is 5.8 mg/dL. I do not have Celiac disease. I have no allergies. My favorite cuisine is Taiwanese cuisine. My daily food budget is $11.5. I am not experiencing food insecurity. I am not a vegetarian. Can you help me with meal planning? Look it up and please provide a detailed meal plan that includes portion sizes. Please make sure to include a list of how much protein, calories, fiber, sodium, phosphorus, calcium, and potassium are in each meal, as well as the daily totals. Please only include a meal plan for a single day. |
| 26 | I am a 67-year-old Hispanic woman on hemodialysis three times a week. My dry weight is 83 kg and my BMI is 30.1. My dietitian recommends I eat a diet with 106 g of protein, 2700 calories, and 25 g of fiber per day. She also recommends no more than 900 mg of phosphorus, no more than 850 mg of calcium, no more than 1850 mg of sodium, and no more than 2950 mg per day of potassium. She also recommends drinking no more than 1300 mL of fluids per day. My albumin is 3.4g/dL and my phosphorus is 5.7 mg/dL. I do not have Celiac disease. I have no allergies. My favorite cuisine is Japanese cuisine. My daily food budget is $13. I am not experiencing food insecurity. I am not a vegetarian. I have high blood pressure. I have diabetes. Can you help me with meal planning? Look it up and please provide a detailed meal plan that includes portion sizes. Please make sure to include a list of how much protein, calories, fiber, sodium, phosphorus, calcium, and potassium are in each meal, as well as the daily totals. Please only include a meal plan for a single day. |
| 27 | I am a 48-year-old Black man on hemodialysis three times a week. My dry weight is 93 kg and my BMI is 31.6. My dietitian recommends I eat a diet with 112 g of protein, 3000 calories, and 24 g of fiber per day. She also recommends no more than 1150 mg of phosphorus, no more than 950 mg of calcium, no more than 2000 mg of sodium, and no more than 2400 mg per day of potassium. She also recommends drinking no more than 1100 mL of fluids per day. My albumin is 3.3g/dL and my phosphorus is 5.4 mg/dL. I do not have Celiac disease. I have no allergies. My favorite cuisine is Argentinian cuisine. My daily food budget is $17. I am experiencing food insecurity. I am not a vegetarian. I have high blood pressure. Can you help me with meal planning? Look it up and please provide a detailed meal plan that includes portion sizes. Please make sure to include a list of how much protein, calories, fiber, sodium, phosphorus, calcium, and potassium are in each meal, as well as the daily totals. Please only include a meal plan for a single day. |
| 28 | I am a 51-year-old Black woman on hemodialysis three times a week. My dry weight is 91 kg and my BMI is 31.3. My dietitian recommends I eat a diet with 85 g of protein, 2300 calories, and 22 g of fiber per day. She also recommends no more than 1000 mg of phosphorus, no more than 900 mg of calcium, no more than 2000 mg of sodium, and no more than 2450 mg per day of potassium. She also recommends drinking no more than 1200 mL of fluids per day. My albumin is 3.3g/dL and my phosphorus is 4.2 mg/dL. I do not have Celiac disease. I have no allergies. My favorite cuisine is Australian cuisine. My daily food budget is $11. I am not experiencing food insecurity. I am not a vegetarian. I have heart failure. I have high blood pressure. I have diabetes. Can you help me with meal planning? Look it up and please provide a detailed meal plan that includes portion sizes. Please make sure to include a list of how much protein, calories, fiber, sodium, phosphorus, calcium, and potassium are in each meal, as well as the daily totals. Please only include a meal plan for a single day. |
| 29 | I am a 52-year-old White man on hemodialysis three times a week. My dry weight is 90 kg and my BMI is 32.7. My dietitian recommends I eat a diet with 109 g of protein, 2950 calories, and 23 g of fiber per day. She also recommends no more than 1300 mg of phosphorus, no more than 1000 mg of calcium, no more than 2000 mg of sodium, and no more than 2600 mg per day of potassium. She also recommends drinking no more than 1200 mL of fluids per day. My albumin is 2.9g/dL and my phosphorus is 3.5 mg/dL. I do not have Celiac disease. I have no allergies. My favorite cuisine is Chinese cuisine. My daily food budget is $18. I am not experiencing food insecurity. I am not a vegetarian. I have high blood pressure. I have diabetes. Can you help me with meal planning? Look it up and please provide a detailed meal plan that includes portion sizes. Please make sure to include a list of how much protein, calories, fiber, sodium, phosphorus, calcium, and potassium are in each meal, as well as the daily totals. Please only include a meal plan for a single day. |
| 30 | I am a 77-year-old White man on hemodialysis three times a week. My dry weight is 77 kg and my BMI is 28.1. My dietitian recommends I eat a diet with 95 g of protein, 2350 calories, and 23 g of fiber per day. She also recommends no more than 1000 mg of phosphorus, no more than 900 mg of calcium, no more than 2000 mg of sodium, and no more than 2950 mg per day of potassium. She also recommends drinking no more than 1100 mL of fluids per day. My albumin is 3.2g/dL and my phosphorus is 7 mg/dL. I do not have Celiac disease. I have no allergies. I have no food preferences. My daily food budget is $13. I am not experiencing food insecurity. I am not a vegetarian. I have high blood pressure. Can you help me with meal planning? Look it up and please provide a detailed meal plan that includes portion sizes. Please make sure to include a list of how much protein, calories, fiber, sodium, phosphorus, calcium, and potassium are in each meal, as well as the daily totals. Please only include a meal plan for a single day. |
| 31 | I am a 23-year-old White man on hemodialysis three times a week. My dry weight is 77 kg and my BMI is 28.8. My dietitian recommends I eat a diet with 94 g of protein, 2550 calories, and 21 g of fiber per day. She also recommends no more than 1000 mg of phosphorus, no more than 900 mg of calcium, no more than 1800 mg of sodium, and no more than 2700 mg per day of potassium. She also recommends drinking no more than 1200 mL of fluids per day. My albumin is 3.2g/dL and my phosphorus is 5.6 mg/dL. I do not have Celiac disease. I have no allergies. I have no food preferences. My daily food budget is $12. I am not experiencing food insecurity. I am not a vegetarian. Can you help me with meal planning? Look it up and please provide a detailed meal plan that includes portion sizes. Please make sure to include a list of how much protein, calories, fiber, sodium, phosphorus, calcium, and potassium are in each meal, as well as the daily totals. Please only include a meal plan for a single day. |
| 32 | I am a 58-year-old Asian man on hemodialysis three times a week. My dry weight is 87 kg and my BMI is 29.3. My dietitian recommends I eat a diet with 91 g of protein, 2450 calories, and 23 g of fiber per day. She also recommends no more than 1000 mg of phosphorus, no more than 900 mg of calcium, no more than 1850 mg of sodium, and no more than 2750 mg per day of potassium. She also recommends drinking no more than 1200 mL of fluids per day. My albumin is 3.4g/dL and my phosphorus is 3.8 mg/dL. I do not have Celiac disease. I have no allergies. My favorite cuisine is Caribbean cuisine. My daily food budget is $12. I am not experiencing food insecurity. I am not a vegetarian. I have high blood pressure. Can you help me with meal planning? Look it up and please provide a detailed meal plan that includes portion sizes. Please make sure to include a list of how much protein, calories, fiber, sodium, phosphorus, calcium, and potassium are in each meal, as well as the daily totals. Please only include a meal plan for a single day. |
| 33 | I am a 48-year-old Black woman on hemodialysis three times a week. My dry weight is 88 kg and my BMI is 32.2. My dietitian recommends I eat a diet with 84 g of protein, 2250 calories, and 21 g of fiber per day. She also recommends no more than 850 mg of phosphorus, no more than 900 mg of calcium, no more than 2000 mg of sodium, and no more than 2800 mg per day of potassium. She also recommends drinking no more than 1100 mL of fluids per day. My albumin is 3g/dL and my phosphorus is 3.5 mg/dL. I do not have Celiac disease. I have no allergies. I have no food preferences. My daily food budget is $11. I am not experiencing food insecurity. I am not a vegetarian. I have high blood pressure. I have diabetes. Can you help me with meal planning? Look it up and please provide a detailed meal plan that includes portion sizes. Please make sure to include a list of how much protein, calories, fiber, sodium, phosphorus, calcium, and potassium are in each meal, as well as the daily totals. Please only include a meal plan for a single day. |
| 34 | I am a 39-year-old Black man on hemodialysis three times a week. My dry weight is 90 kg and my BMI is 31.1. My dietitian recommends I eat a diet with 90 g of protein, 2450 calories, and 21 g of fiber per day. She also recommends no more than 900 mg of phosphorus, no more than 800 mg of calcium, no more than 2000 mg of sodium, and no more than 2600 mg per day of potassium. She also recommends drinking no more than 1100 mL of fluids per day. My albumin is 3.5g/dL and my phosphorus is 4.7 mg/dL. I do not have Celiac disease. I am allergic to peanuts. I have no food preferences. My daily food budget is $11. I am experiencing food insecurity. I am not a vegetarian. Can you help me with meal planning? Look it up and please provide a detailed meal plan that includes portion sizes. Please make sure to include a list of how much protein, calories, fiber, sodium, phosphorus, calcium, and potassium are in each meal, as well as the daily totals. Please only include a meal plan for a single day. |
| 35 | I am a 69-year-old White man on hemodialysis three times a week. My dry weight is 88 kg and my BMI is 30. My dietitian recommends I eat a diet with 100 g of protein, 2300 calories, and 23 g of fiber per day. She also recommends no more than 1000 mg of phosphorus, no more than 1000 mg of calcium, no more than 1850 mg of sodium, and no more than 3100 mg per day of potassium. She also recommends drinking no more than 1300 mL of fluids per day. My albumin is 3.1g/dL and my phosphorus is 4.7 mg/dL. I do not have Celiac disease. I have no allergies. My favorite cuisine is Mexican cuisine. My daily food budget is $12.5. I am not experiencing food insecurity. I am not a vegetarian. I have high blood pressure. Can you help me with meal planning? Look it up and please provide a detailed meal plan that includes portion sizes. Please make sure to include a list of how much protein, calories, fiber, sodium, phosphorus, calcium, and potassium are in each meal, as well as the daily totals. Please only include a meal plan for a single day. |
| 36 | I am a 62-year-old Hispanic man on hemodialysis three times a week. My dry weight is 85 kg and my BMI is 30.6. My dietitian recommends I eat a diet with 96 g of protein, 2550 calories, and 22 g of fiber per day. She also recommends no more than 1050 mg of phosphorus, no more than 850 mg of calcium, no more than 1800 mg of sodium, and no more than 2550 mg per day of potassium. She also recommends drinking no more than 1200 mL of fluids per day. My albumin is 3.5g/dL and my phosphorus is 5.2 mg/dL. I do not have Celiac disease. I have no allergies. My favorite cuisine is Danish cuisine. My daily food budget is $12. I am not experiencing food insecurity. I am not a vegetarian. I have high blood pressure. I have diabetes. Can you help me with meal planning? Look it up and please provide a detailed meal plan that includes portion sizes. Please make sure to include a list of how much protein, calories, fiber, sodium, phosphorus, calcium, and potassium are in each meal, as well as the daily totals. Please only include a meal plan for a single day. |
| 37 | I am a 72-year-old Hispanic woman on hemodialysis three times a week. My dry weight is 85 kg and my BMI is 28.7. My dietitian recommends I eat a diet with 106 g of protein, 2650 calories, and 22 g of fiber per day. She also recommends no more than 800 mg of phosphorus, no more than 1000 mg of calcium, no more than 1800 mg of sodium, and no more than 2850 mg per day of potassium. She also recommends drinking no more than 1200 mL of fluids per day. My albumin is 2.9g/dL and my phosphorus is 6.4 mg/dL. I do not have Celiac disease. I have no allergies. My favorite cuisine is Vietnamese cuisine. My daily food budget is $11.5. I am not experiencing food insecurity. I am not a vegetarian. I have heart failure. I have high blood pressure. I have diabetes. Can you help me with meal planning? Look it up and please provide a detailed meal plan that includes portion sizes. Please make sure to include a list of how much protein, calories, fiber, sodium, phosphorus, calcium, and potassium are in each meal, as well as the daily totals. Please only include a meal plan for a single day. |
| 38 | I am a 61-year-old Black man on hemodialysis three times a week. My dry weight is 82 kg and my BMI is 29.9. My dietitian recommends I eat a diet with 86 g of protein, 2150 calories, and 21 g of fiber per day. She also recommends no more than 900 mg of phosphorus, no more than 1000 mg of calcium, no more than 2000 mg of sodium, and no more than 2300 mg per day of potassium. She also recommends drinking no more than 1300 mL of fluids per day. My albumin is 2.8g/dL and my phosphorus is 3.3 mg/dL. I do not have Celiac disease. I have no allergies. My favorite cuisine is Norwegian cuisine. My daily food budget is $16.5. I am not experiencing food insecurity. I am not a vegetarian. I have high blood pressure. I have diabetes. Can you help me with meal planning? Look it up and please provide a detailed meal plan that includes portion sizes. Please make sure to include a list of how much protein, calories, fiber, sodium, phosphorus, calcium, and potassium are in each meal, as well as the daily totals. Please only include a meal plan for a single day. |
| 39 | I am a 67-year-old Black man on hemodialysis three times a week. My dry weight is 85 kg and my BMI is 28.8. My dietitian recommends I eat a diet with 101 g of protein, 2500 calories, and 23 g of fiber per day. She also recommends no more than 950 mg of phosphorus, no more than 1000 mg of calcium, no more than 2000 mg of sodium, and no more than 2350 mg per day of potassium. She also recommends drinking no more than 1000 mL of fluids per day. My albumin is 4.2g/dL and my phosphorus is 6.9 mg/dL. I do not have Celiac disease. I have no allergies. My favorite cuisine is Danish cuisine. My daily food budget is $12. I am experiencing food insecurity. I am not a vegetarian. I have heart failure. I have high blood pressure. I have diabetes. Can you help me with meal planning? Look it up and please provide a detailed meal plan that includes portion sizes. Please make sure to include a list of how much protein, calories, fiber, sodium, phosphorus, calcium, and potassium are in each meal, as well as the daily totals. Please only include a meal plan for a single day. |
| 40 | I am a 61-year-old Black man on hemodialysis three times a week. My dry weight is 88 kg and my BMI is 29.1. My dietitian recommends I eat a diet with 103 g of protein, 2500 calories, and 23 g of fiber per day. She also recommends no more than 1000 mg of phosphorus, no more than 850 mg of calcium, no more than 1900 mg of sodium, and no more than 2600 mg per day of potassium. She also recommends drinking no more than 1100 mL of fluids per day. My albumin is 3.1g/dL and my phosphorus is 5.6 mg/dL. I do not have Celiac disease. I have no allergies. I have no food preferences. My daily food budget is $13. I am not experiencing food insecurity. I am not a vegetarian. I have heart failure. I have high blood pressure. Can you help me with meal planning? Look it up and please provide a detailed meal plan that includes portion sizes. Please make sure to include a list of how much protein, calories, fiber, sodium, phosphorus, calcium, and potassium are in each meal, as well as the daily totals. Please only include a meal plan for a single day. |
| 41 | I am a 75-year-old White man on hemodialysis three times a week. My dry weight is 81 kg and my BMI is 28.6. My dietitian recommends I eat a diet with 88 g of protein, 2150 calories, and 23 g of fiber per day. She also recommends no more than 950 mg of phosphorus, no more than 1000 mg of calcium, no more than 2000 mg of sodium, and no more than 2600 mg per day of potassium. She also recommends drinking no more than 1100 mL of fluids per day. My albumin is 3.3g/dL and my phosphorus is 5 mg/dL. I do not have Celiac disease. I have no allergies. My favorite cuisine is Danish cuisine. My daily food budget is $11.5. I am not experiencing food insecurity. I am not a vegetarian. I have high blood pressure. Can you help me with meal planning? Look it up and please provide a detailed meal plan that includes portion sizes. Please make sure to include a list of how much protein, calories, fiber, sodium, phosphorus, calcium, and potassium are in each meal, as well as the daily totals. Please only include a meal plan for a single day. |
| 42 | I am a 63-year-old Black woman on hemodialysis three times a week. My dry weight is 87 kg and my BMI is 30.6. My dietitian recommends I eat a diet with 90 g of protein, 2200 calories, and 21 g of fiber per day. She also recommends no more than 1050 mg of phosphorus, no more than 1000 mg of calcium, no more than 1700 mg of sodium, and no more than 2550 mg per day of potassium. She also recommends drinking no more than 1100 mL of fluids per day. My albumin is 3.4g/dL and my phosphorus is 5.5 mg/dL. I do not have Celiac disease. I have no allergies. I have no food preferences. My daily food budget is $14. I am not experiencing food insecurity. I am not a vegetarian. I have high blood pressure. I have diabetes. Can you help me with meal planning? Look it up and please provide a detailed meal plan that includes portion sizes. Please make sure to include a list of how much protein, calories, fiber, sodium, phosphorus, calcium, and potassium are in each meal, as well as the daily totals. Please only include a meal plan for a single day. |
| 43 | I am a 62-year-old Black woman on hemodialysis three times a week. My dry weight is 85 kg and my BMI is 30.3. My dietitian recommends I eat a diet with 89 g of protein, 2100 calories, and 24 g of fiber per day. She also recommends no more than 900 mg of phosphorus, no more than 1000 mg of calcium, no more than 1750 mg of sodium, and no more than 2600 mg per day of potassium. She also recommends drinking no more than 1400 mL of fluids per day. My albumin is 3.1g/dL and my phosphorus is 4.1 mg/dL. I do not have Celiac disease. I have no allergies. I have no food preferences. My daily food budget is $12. I am experiencing food insecurity. I am not a vegetarian. I have high blood pressure. I have diabetes. Can you help me with meal planning? Look it up and please provide a detailed meal plan that includes portion sizes. Please make sure to include a list of how much protein, calories, fiber, sodium, phosphorus, calcium, and potassium are in each meal, as well as the daily totals. Please only include a meal plan for a single day. |
| 44 | I am a 66-year-old Black woman on hemodialysis three times a week. My dry weight is 82 kg and my BMI is 30.7. My dietitian recommends I eat a diet with 87 g of protein, 2350 calories, and 24 g of fiber per day. She also recommends no more than 850 mg of phosphorus, no more than 1000 mg of calcium, no more than 2000 mg of sodium, and no more than 2650 mg per day of potassium. She also recommends drinking no more than 1200 mL of fluids per day. My albumin is 3.4g/dL and my phosphorus is 6.4 mg/dL. I do not have Celiac disease. I have no allergies. I have no food preferences. My daily food budget is $12. I am not experiencing food insecurity. I am not a vegetarian. I have high blood pressure. Can you help me with meal planning? Look it up and please provide a detailed meal plan that includes portion sizes. Please make sure to include a list of how much protein, calories, fiber, sodium, phosphorus, calcium, and potassium are in each meal, as well as the daily totals. Please only include a meal plan for a single day. |
| 45 | I am a 76-year-old White man on hemodialysis three times a week. My dry weight is 77 kg and my BMI is 30.4. My dietitian recommends I eat a diet with 88 g of protein, 2200 calories, and 23 g of fiber per day. She also recommends no more than 850 mg of phosphorus, no more than 1000 mg of calcium, no more than 2000 mg of sodium, and no more than 2900 mg per day of potassium. She also recommends drinking no more than 1100 mL of fluids per day. My albumin is 3.3g/dL and my phosphorus is 6 mg/dL. I do not have Celiac disease. I have no allergies. My favorite cuisine is Singaporean cuisine. My daily food budget is $13.5. I am not experiencing food insecurity. I am not a vegetarian. I have heart failure. I have high blood pressure. I have diabetes. Can you help me with meal planning? Look it up and please provide a detailed meal plan that includes portion sizes. Please make sure to include a list of how much protein, calories, fiber, sodium, phosphorus, calcium, and potassium are in each meal, as well as the daily totals. Please only include a meal plan for a single day. |
| 46 | I am a 77-year-old White man on hemodialysis three times a week. My dry weight is 80 kg and my BMI is 27.8. My dietitian recommends I eat a diet with 99 g of protein, 2500 calories, and 23 g of fiber per day. She also recommends no more than 1100 mg of phosphorus, no more than 950 mg of calcium, no more than 2000 mg of sodium, and no more than 2450 mg per day of potassium. She also recommends drinking no more than 1200 mL of fluids per day. My albumin is 3.1g/dL and my phosphorus is 3.4 mg/dL. I do not have Celiac disease. I have no allergies. I have no food preferences. My daily food budget is $12. I am not experiencing food insecurity. I am not a vegetarian. I have heart failure. I have high blood pressure. Can you help me with meal planning? Look it up and please provide a detailed meal plan that includes portion sizes. Please make sure to include a list of how much protein, calories, fiber, sodium, phosphorus, calcium, and potassium are in each meal, as well as the daily totals. Please only include a meal plan for a single day. |
| 47 | I am a 61-year-old Black man on hemodialysis three times a week. My dry weight is 84 kg and my BMI is 27.9. My dietitian recommends I eat a diet with 109 g of protein, 2750 calories, and 23 g of fiber per day. She also recommends no more than 1150 mg of phosphorus, no more than 1000 mg of calcium, no more than 2000 mg of sodium, and no more than 2850 mg per day of potassium. She also recommends drinking no more than 1000 mL of fluids per day. My albumin is 3.2g/dL and my phosphorus is 4 mg/dL. I do not have Celiac disease. I have no allergies. My favorite cuisine is Danish cuisine. My daily food budget is $11.5. I am not experiencing food insecurity. I am not a vegetarian. I have high blood pressure. I have diabetes. Can you help me with meal planning? Look it up and please provide a detailed meal plan that includes portion sizes. Please make sure to include a list of how much protein, calories, fiber, sodium, phosphorus, calcium, and potassium are in each meal, as well as the daily totals. Please only include a meal plan for a single day. |
| 48 | I am a 70-year-old Black woman on hemodialysis three times a week. My dry weight is 81 kg and my BMI is 30.2. My dietitian recommends I eat a diet with 87 g of protein, 2300 calories, and 23 g of fiber per day. She also recommends no more than 900 mg of phosphorus, no more than 950 mg of calcium, no more than 2000 mg of sodium, and no more than 2600 mg per day of potassium. She also recommends drinking no more than 1100 mL of fluids per day. My albumin is 3.2g/dL and my phosphorus is 4.9 mg/dL. I do not have Celiac disease. I have no allergies. My favorite cuisine is Turkish cuisine. My daily food budget is $12.5. I am not experiencing food insecurity. I am not a vegetarian. I have heart failure. I have high blood pressure. I have diabetes. Can you help me with meal planning? Look it up and please provide a detailed meal plan that includes portion sizes. Please make sure to include a list of how much protein, calories, fiber, sodium, phosphorus, calcium, and potassium are in each meal, as well as the daily totals. Please only include a meal plan for a single day. |
| 49 | I am a 57-year-old Black man on hemodialysis three times a week. My dry weight is 88 kg and my BMI is 30.1. My dietitian recommends I eat a diet with 100 g of protein, 2700 calories, and 23 g of fiber per day. She also recommends no more than 1150 mg of phosphorus, no more than 1000 mg of calcium, no more than 2000 mg of sodium, and no more than 2900 mg per day of potassium. She also recommends drinking no more than 1100 mL of fluids per day. My albumin is 3.8g/dL and my phosphorus is 4 mg/dL. I do not have Celiac disease. I have no allergies. I have no food preferences. My daily food budget is $14.5. I am not experiencing food insecurity. I am not a vegetarian. I have heart failure. I have high blood pressure. Can you help me with meal planning? Look it up and please provide a detailed meal plan that includes portion sizes. Please make sure to include a list of how much protein, calories, fiber, sodium, phosphorus, calcium, and potassium are in each meal, as well as the daily totals. Please only include a meal plan for a single day. |
| 50 | I am a 86-year-old Black woman on hemodialysis three times a week. My dry weight is 77 kg and my BMI is 25.1. My dietitian recommends I eat a diet with 82 g of protein, 1950 calories, and 23 g of fiber per day. She also recommends no more than 900 mg of phosphorus, no more than 900 mg of calcium, no more than 2000 mg of sodium, and no more than 2150 mg per day of potassium. She also recommends drinking no more than 1200 mL of fluids per day. My albumin is 3g/dL and my phosphorus is 3.1 mg/dL. I do not have Celiac disease. I have no allergies. My favorite cuisine is Moroccan cuisine. My daily food budget is $11. I am not experiencing food insecurity. I am not a vegetarian. I have high blood pressure. Can you help me with meal planning? Look it up and please provide a detailed meal plan that includes portion sizes. Please make sure to include a list of how much protein, calories, fiber, sodium, phosphorus, calcium, and potassium are in each meal, as well as the daily totals. Please only include a meal plan for a single day. |
