# Supplementary material for: Assessment of large language model chatbots for hemodialysis meal planning: a descriptive study
Source: BMC Nephrol. 2026 Mar 31;27:298. doi: 10.1186/s12882-026-04936-8 (PMC13162381; doi:10.1186/s12882-026-04936-8)
Supplement: Supplementary file 2 — Supplementary Material 2 [file 12882_2026_4936_MOESM2_ESM.docx]

Supplement S2 – Example LLM responses to a prompt #1 across ChatGPT, Claude, Llama and Gemini. Entries were copied and pasted from each LLM’s respective web-interface without modification or reformatting.

**Prompt 1:**

I am a 53-year-old White man on hemodialysis three times a week. My dry weight is 94 kg and my BMI is 29.6. My dietitian recommends I eat a diet with 116 g of protein, 3100 calories, and 19 g of fiber per day. She also recommends no more than 1250 mg of phosphorus, no more than 900 mg of calcium, no more than 1900 mg of sodium, and no more than 2650 mg per day of potassium. She also recommends drinking no more than 1200 mL of fluids per day. My albumin is 3g/dL and my phosphorus is 4.7 mg/dL. I do not have Celiac disease. I have no allergies. My favorite cuisine is French cuisine. My daily food budget is $12.5. I am not experiencing food insecurity. I am not a vegetarian. I have heart failure. I have diabetes. Can you help me with meal planning? Look it up and please provide a detailed meal plan that includes portion sizes. Please make sure to include a list of how much protein, calories, fiber, sodium, phosphorus, calcium, and potassium are in each meal, as well as the daily totals. Please only include a meal plan for a single day.

**ChatGPT Response**

Here’s a French‐inspired one‐day menu tailored to your dialysis, heart‐failure and diabetic needs, aiming for ~116 g protein, ~3 100 kcal, ≥19 g fiber, ≤1 250 mg phosphorus, ≤900 mg calcium, ≤1 900 mg sodium, ≤2 650 mg potassium, and ≤1 200 mL fluids. All values are approximate and from USDA‐based sources—please adjust slightly to hit your exact targets.

| **Meal** | **Food & Portion** | **Calories (kcal)** | **Protein (g)** | **Fiber (g)** | **Sodium (mg)** | **Phosphorus (mg)** | **Calcium (mg)** | **Potassium (mg)** |
| --- | --- | --- | --- | --- | --- | --- | --- | --- |
| **Breakfast** | 3 large eggs (150 g)  + 1 tsp grated Parmesan (1.7 g)  + 1 tsp salted butter (5 g)  + 1 slice French bread (50 g)  + 1 butter croissant (60 g) | 624 | 28.8 | 3.0 | 995 | 423 | 162 | 334 |
| **Snack 1** | 100 g plain whole‑milk Greek yogurt  + 1 medium banana (118 g) | 194 | 10.1 | 5.6 | 36 | 157 | 106 | 563 |
| **Lunch** | 130 g roasted chicken breast  + 1 cup cooked white rice (205 g)  + 1 cup ratatouille (214 g) | 527 | 45.7 | 4.8 | 606 | 464 | 79 | 1 020 |
| **Snack 2** | 2 slices French bread (100 g)  + 1 Tbsp peanut butter (16 g) [eatthismuch.com](https://www.eatthismuch.com/calories/peanut-butter-3640) | 464 | 12.4 | 2.5 | 460 | 178 | 54 | 264 |
| **Dinner** | 150 g Atlantic cod, baked  + 1 cup cooked green beans (125 g)  + ½ cup cooked carrots (65 g) | 303 | 36.9 | 5.2 | 140 | 255 | 91 | 894 |
| **Snack 3** | 1 medium apple (182 g)  + 1 oz cheddar cheese (28 g) | 208 | 7.5 | 4.4 | 182 | 165 | 211 | 215 |
| **Daily Totals** |  | **2 320 kcal** | **141 g** | **25 g** | **2 419 mg** | **1 642 mg** | **703 mg** | **3 290 mg** |

**How this meets (and where to tweak) your targets:**

- **Protein (goal 116 g):** ~141 g – you can shave ~25 g by reducing chicken to 100 g (–9 g) and cod to 100 g (–11 g) or cutting back peanut butter.
- **Calories (goal 3 100 kcal):** ~2 320 kcal – add 1 Tbsp olive oil (≈102 kcal), a handful of almonds (30 g ≈174 kcal), and/or increase rice at lunch to 1.5 cups (+97 kcal) to hit ~3 100 kcal.
- **Fiber (goal ≥19 g):** ~25 g – on target.
- **Sodium (≤1 900 mg):** ~2 419 mg – trim by choosing low‑sodium bread, skipping croissant or peanut butter, or using unsalted butter.
- **Phosphorus (≤1 250 mg):** ~1 642 mg – lower by reducing cheese, peanut butter, or yogurt portions.
- **Calcium (≤900 mg):** ~703 mg – on target.
- **Potassium (≤2 650 mg):** ~3 290 mg – reduce banana, green beans, or apple to bring it down.

**Next steps:** Adjust portion sizes of the higher‐mineral items (banana, green beans, yogurt, peanut butter) or swap to lower‐potassium alternatives (e.g. grapes, strawberries) and use lower‐sodium breads/spreads. This template gives you a French‐style framework—fine‐tune the exact weights to land squarely on your prescribed targets.

**Claude Response**

I'd be happy to help you create a meal plan that meets your dietary requirements while accommodating your medical conditions and preferences for French cuisine. Since you're on hemodialysis and have both heart failure and diabetes, this plan carefully balances your nutritional needs while staying within your budget.

**One-Day Meal Plan**

**Breakfast**

- 2 scrambled eggs (30g protein, 180 calories)
- 1 slice sourdough toast (4g protein, 80 calories)
- 1 tsp unsalted butter (0g protein, 35 calories)
- 1/2 cup blueberries (0.5g protein, 40 calories)
- 1 cup coffee with 2 tbsp non-dairy creamer (0g protein, 40 calories)

*Breakfast Nutrition: 34.5g protein, 375 calories, 2g fiber, 180mg sodium, 230mg phosphorus, 80mg calcium, 300mg potassium*

**Morning Snack**

- 1 medium apple (0.5g protein, 95 calories)
- 1 oz (28g) soft goat cheese (6g protein, 80 calories)

*Morning Snack Nutrition: 6.5g protein, 175 calories, 4g fiber, 75mg sodium, 90mg phosphorus, 40mg calcium, 200mg potassium*

**Lunch**

- 3 oz (85g) roasted chicken breast (26g protein, 140 calories)
- 1 cup white rice (4g protein, 200 calories)
- 1/2 cup green beans sautéed in olive oil (1g protein, 70 calories)
- 1 small dinner roll (3g protein, 85 calories)
- 1 tsp unsalted butter (0g protein, 35 calories)
- 1/2 cup cranberry juice diluted with water (0g protein, 70 calories)

*Lunch Nutrition: 34g protein, 600 calories, 3g fiber, 290mg sodium, 245mg phosphorus, 120mg calcium, 420mg potassium*

**Afternoon Snack**

- 1/2 cup strawberries (0.5g protein, 25 calories)
- 1/4 cup whipped cream (1g protein, 100 calories)

*Afternoon Snack Nutrition: 1.5g protein, 125 calories, 1.5g fiber, 15mg sodium, 30mg phosphorus, 60mg calcium, 150mg potassium*

**Dinner**

- 4 oz (115g) baked salmon (23g protein, 230 calories)
- 3/4 cup mashed potatoes (3g protein, 150 calories)
- 1 tbsp butter (0g protein, 100 calories)
- 1/2 cup steamed carrots (1g protein, 25 calories)
- 1/2 cup cooked leeks (1g protein, 15 calories)
- 1 small dinner roll (3g protein, 85 calories)
- 1 tsp butter (0g protein, 35 calories)

*Dinner Nutrition: 31g protein, 640 calories, 5g fiber, 300mg sodium, 350mg phosphorus, 120mg calcium, 780mg potassium*

**Evening Snack**

- 1 cup vanilla rice pudding made with rice milk (8g protein, 240 calories)
- 1 tbsp honey (0g protein, 60 calories)
- 2 shortbread cookies (1g protein, 140 calories)

*Evening Snack Nutrition: 9g protein, 440 calories, 1g fiber, 180mg sodium, 110mg phosphorus, 60mg calcium, 220mg potassium*

**Daily Water/Fluids**

- 1000 mL total for the day (includes coffee, juice, and water with meals)

**Daily Nutrition Totals**

- **Protein:** 116.5g (target: 116g) ✓
- **Calories:** 2,355 calories (target: 3100 calories) - below target but can be adjusted with additional fats
- **Fiber:** 16.5g (target: 19g) - slightly below target
- **Sodium:** 1,040mg (target: max 1900mg) ✓
- **Phosphorus:** 1,055mg (target: max 1250mg) ✓
- **Calcium:** 480mg (target: max 900mg) ✓
- **Potassium:** 2,070mg (target: max 2650mg) ✓
- **Fluids:** 1000mL (target: max 1200mL) ✓
- **Cost:** Approximately $11.75 per day (within your $12.50 budget)

**Key considerations for this meal plan:**

1. **Protein sources** are chosen to help improve your albumin level (3g/dL is lower than ideal).
2. **Lower phosphorus foods** are emphasized since your phosphorus level (4.7mg/dL) is already in the upper range.
3. **French cuisine influences** are incorporated through preparation styles and food combinations.
4. **Diabetes management** is addressed with moderate carbohydrates and balanced meals.
5. **Heart failure considerations** are met with the low sodium content.
6. **Fluid restriction** is carefully managed within your 1200mL limit.

Would you like me to adjust anything in this meal plan to better suit your preferences?

**Gemini Response**

Okay, I can help you create a one-day French-inspired meal plan that aims to meet your dietitian's recommendations while considering your medical conditions and preferences. Please remember that this is just a sample meal plan, and it's essential to consult with your dietitian for personalized guidance and adjustments.

Here is a potential meal plan for a single day:

**French-Inspired Meal Plan**

**Breakfast (Approximately 425 calories, 35g protein, 4g fiber, 180mg sodium, 280mg phosphorus, 150mg calcium, 450mg potassium)**

- **Oeufs Brouillés aux Fines Herbes (Scrambled Egg Whites with Herbs):**
  - Portion: 6 large egg whites
  - Protein: ~20g
  - Calories: ~100 kcal
  - Fiber: ~0g
  - Sodium: ~80mg
  - Phosphorus: ~20mg
  - Calcium: ~20mg
  - Potassium: ~80mg
  - Preparation: Whisk egg whites with 1 tablespoon of chopped fresh herbs (such as parsley, chives, and tarragon). Cook in a non-stick pan with a small amount of olive oil spray until set.
- **Petite Tranche de Pain Complet Grillé (Small Slice of Toasted Whole Wheat Bread):**
  - Portion: 1 slice (approximately 30g)
  - Protein: ~2g
  - Calories: ~75 kcal
  - Fiber: ~2g
  - Sodium: ~70mg
  - Phosphorus: ~60mg
  - Calcium: ~15mg
  - Potassium: ~70mg
- **Beurre Léger (Light Butter):**
  - Portion: 1 teaspoon (5g)
  - Protein: ~0g
  - Calories: ~35 kcal
  - Fiber: ~0g
  - Sodium: ~0mg
  - Phosphorus: ~0mg
  - Calcium: ~0mg
  - Potassium: ~0mg
- **Compote de Fruits Rouges (Small Berry Compote):**
  - Portion: ½ cup (approximately 75g) of mixed berries (strawberries, blueberries, raspberries)
  - Protein: ~1g
  - Calories: ~45 kcal
  - Fiber: ~2g
  - Sodium: ~1mg
  - Phosphorus: ~15mg
  - Calcium: ~10mg
  - Potassium: ~100mg
  - Preparation: Gently heat the berries in a saucepan with a tiny amount of water until slightly softened. No added sugar.
- **Café au Lait (Coffee with Low-Phosphorus Milk Alternative):**
  - Portion: ½ cup brewed coffee with ¼ cup unsweetened almond milk
  - Protein: ~1g
  - Calories: ~50 kcal
  - Fiber: ~0g
  - Sodium: ~30mg
  - Phosphorus: ~185mg
  - Calcium: ~95mg
  - Potassium: ~100mg

**Lunch (Approximately 850 calories, 45g protein, 6g fiber, 450mg sodium, 350mg phosphorus, 180mg calcium, 700mg potassium)**

- **Salade Niçoise Modifiée (Modified Niçoise Salad):**
  - Portion: Approximately 2 cups
  - Protein: ~30g
  - Calories: ~550 kcal
  - Fiber: ~4g
  - Sodium: ~300mg
  - Phosphorus: ~200mg
  - Calcium: ~100mg
  - Potassium: ~400mg
  - Ingredients:
    - 3 oz canned light tuna in water, drained
    - 1 cup chopped romaine lettuce
    - ½ cup steamed and cooled green beans (lower in potassium than raw)
    - ¼ cup sliced cucumber
    - ¼ cup chopped red bell pepper
    - 1 hard-boiled egg, sliced (limit to one due to phosphorus)
    - 2 small Kalamata olives, halved (limit due to sodium)
    - 1 tablespoon low-sodium vinaigrette dressing (homemade with olive oil, vinegar, herbs)
- **Petite Baguette (Small Baguette):**
  - Portion: ½ small baguette (approximately 75g)
  - Protein: ~7g
  - Calories: ~200 kcal
  - Fiber: ~2g
  - Sodium: ~100mg
  - Phosphorus: ~100mg
  - Calcium: ~30mg
  - Potassium: ~200mg
- **Eau (Water):**
  - Portion: 8 fl oz

**Dinner (Approximately 975 calories, 30g protein, 5g fiber, 550mg sodium, 400mg phosphorus, 250mg calcium, 750mg potassium)**

- **Poulet Rôti aux Herbes de Provence (Roasted Chicken with Herbs de Provence):**
  - Portion: 4 oz (approximately 113g) boneless, skinless chicken breast
  - Protein: ~35g
  - Calories: ~165 kcal
  - Fiber: ~0g
  - Sodium: ~60mg
  - Phosphorus: ~200mg
  - Calcium: ~15mg
  - Potassium: ~350mg
  - Preparation: Season chicken breast with Herbs de Provence and a small amount of salt and pepper. Roast or bake until cooked through.
- **Purée de Chou-Fleur (Cauliflower Puree):**
  - Portion: 1 cup (approximately 150g)
  - Protein: ~2g
  - Calories: ~50 kcal
  - Fiber: ~2g
  - Sodium: ~20mg
  - Phosphorus: ~50mg
  - Calcium: ~20mg
  - Potassium: ~250mg
  - Preparation: Steam or boil cauliflower until very tender. Mash or blend with a small amount of unsweetened almond milk, a drizzle of olive oil, and a pinch of nutmeg. Avoid adding cheese or cream.
- **Haricots Verts Vapeur (Steamed Green Beans):**
  - Portion: 1 cup (approximately 100g)
  - Protein: ~1g
  - Calories: ~30 kcal
  - Fiber: ~2g
  - Sodium: ~5mg
  - Phosphorus: ~25mg
  - Calcium: ~30mg
  - Potassium: ~100mg
- **Petite Salade Verte (Small Green Salad):**
  - Portion: 1 cup mixed greens
  - Protein: ~1g
  - Calories: ~15 kcal
  - Fiber: ~1g
  - Sodium: ~5mg
  - Phosphorus: ~10mg
  - Calcium: ~10mg
  - Potassium: ~50mg
  - Dressing: 1 tablespoon low-sodium vinaigrette (homemade)
- **Tarte aux Pommes Modifiée (Modified Apple Tart):**
  - Portion: ¼ of a small tart (using a low-phosphorus crust and limited apples)
  - Protein: ~1g
  - Calories: ~225 kcal
  - Fiber: ~0g
  - Sodium: ~40mg
  - Phosphorus: ~10mg
  - Calcium: ~25mg
  - Potassium: ~0mg (using a very small amount of apple)
  - Preparation: This would require a special recipe using low-phosphorus ingredients for the crust (e.g., using rice flour) and a minimal amount of thinly sliced apple. Sweeten with a non-caloric sweetener. This is challenging within the budget and restrictions and might need to be replaced with a very small portion of another fruit or a different type of low-phosphorus dessert if feasible.
- **Eau (Water):**
  - Portion: 8 fl oz

**Snacks (Approximately 500 calories, 6g protein, 4g fiber, 220mg sodium, 200mg phosphorus, 180mg calcium, 450mg potassium)**

- **Yaourt Grec Nature Allégé (Plain Nonfat Greek Yogurt):**
  - Portion: ½ cup (approximately 150g)
  - Protein: ~13g
  - Calories: ~80 kcal
  - Fiber: ~0g
  - Sodium: ~60mg
  - Phosphorus: ~200mg
  - Calcium: ~200mg
  - Potassium: ~200mg
- **Petite Pomme (Small Apple):**
  - Portion: 1 small apple (approximately 100g)
  - Protein: ~0g
  - Calories: ~50 kcal
  - Fiber: ~2g
  - Sodium: ~1mg
  - Phosphorus: ~10mg
  - Calcium: ~5mg
  - Potassium: ~100mg
- **Craquelins de Riz Non Salés (Unsalted Rice Cakes):**
  - Portion: 3 rice cakes
  - Protein: ~2g
  - Calories: ~100 kcal
  - Fiber: ~2g
  - Sodium: ~10mg
  - Phosphorus: ~20mg
  - Calcium: ~10mg
  - Potassium: ~50mg
- **Amandes Non Salées (Unsalted Almonds):**
  - Portion: ¼ cup (approximately 35g)
  - Protein: ~7g
  - Calories: ~200 kcal
  - Fiber: ~3.5g
  - Sodium: ~1mg
  - Phosphorus: ~135mg
  - Calcium: ~80mg
  - Potassium: ~250mg

**Daily Totals (Estimated):**

- **Calories:** ~2750 kcal (Needs adjustment to reach 3100 kcal - consider adding a small additional snack or increasing portion sizes slightly where appropriate and safe within other limits)
- **Protein:** ~116g
- **Fiber:** ~19g
- **Sodium:** ~1400mg
- **Phosphorus:** ~1230mg
- **Calcium:** ~760mg
- **Potassium:** ~2350mg
- **Fluids:** Approximately 1050 mL (from food and beverages)

**Important Considerations and Budget Notes:**

- **Budget:** This meal plan attempts to use relatively inexpensive ingredients. Buying in bulk when possible and choosing seasonal produce can help stay within the $12.5 budget. Egg whites are generally affordable. Canned tuna is a budget-friendly protein source. Rice cakes and plain yogurt are also usually inexpensive. Almonds can be a bit pricier, so consider smaller portions or alternatives if needed. The apple tart might be challenging to make within the budget with specialized ingredients; consider a simpler fruit option instead.
- **Fluid Restriction:** The estimated fluid intake from this plan is around 1050 mL (including coffee, almond milk, and water). You have a limit of 1200 mL, so this plan leaves some room for additional fluids if needed, but be mindful of this limit.
- **Phosphorus:** The phosphorus content is close to the upper limit. It's crucial to choose low-phosphorus options within each food group. For example, egg whites are much lower in phosphorus than whole eggs.
- **Potassium:** The potassium is within the recommended range. Be mindful of portion sizes for fruits and vegetables. Steaming vegetables can help reduce potassium content.
- **Sodium:** The sodium is well within the limit. Using fresh herbs and making your own low-sodium dressings is helpful. Limit processed foods and canned goods with added sodium.
- **Calcium:** The calcium is within the recommended range.
- **Protein:** The protein target of 116g is met in this plan.
- **Calories:** The estimated calorie total is around 2750 kcal, which is below the recommended 3100 kcal. You might need to increase portion sizes of some items, particularly those high in healthy fats and protein, or add a small extra snack, while staying within your other nutrient limits. Consider adding a small serving of olive oil with your meals or an additional low-phosphorus, low-potassium snack like a few more unsalted rice cakes with a thin spread of unsalted butter or a small amount of avocado (if potassium allows).
- **Diabetes:** This meal plan includes complex carbohydrates (whole wheat bread, baguette, vegetables) and protein to help manage blood sugar. Be mindful of portion sizes, especially for the baguette and apple. Monitor your blood glucose levels as recommended by your doctor.
- **Heart Failure:** This plan is mindful of sodium and fluid restrictions, which are important for managing heart failure.

**Important Notes:**

- This meal plan is a suggestion and should be reviewed and adjusted by your registered dietitian to ensure it meets your individual needs and medical conditions.
- Nutritional values can vary depending on the specific brands and preparation methods of the foods.
- Always consult with your doctor or dietitian before making significant changes to your diet.
- This meal plan focuses on a single day. Variety is important for a balanced diet, so work with your dietitian to create a rotating menu for your other days.

I hope this detailed meal plan is helpful! Let me know if you have any other questions.

**Llama Response**

Given your dietary requirements and preferences for French cuisine, I've created a meal plan that should meet your needs while staying within your budget. Here's a detailed plan for one day:

**Breakfast**

- **French Toast with Fresh Berries and Yogurt**
  - 2 slices whole wheat bread (140 calories, 4g protein, 4g fiber, 350mg sodium, 120mg phosphorus, 150mg calcium, 200mg potassium)
  - 1 large egg (70 calories, 6g protein, 0g fiber, 70mg sodium, 90mg phosphorus, 25mg calcium, 70mg potassium)
  - 1/2 cup fresh berries (60 calories, 1g protein, 2g fiber, 10mg sodium, 10mg phosphorus, 20mg calcium, 100mg potassium)
  - 1/2 cup low-fat yogurt (50 calories, 5g protein, 0g fiber, 50mg sodium, 80mg phosphorus, 150mg calcium, 200mg potassium)

Total: 320 calories, 16g protein, 6g fiber, 480mg sodium, 300mg phosphorus, 345mg calcium, 570mg potassium

**Lunch**

- **Grilled Chicken Breast with Quinoa and Steamed Broccoli**
  - 3 oz grilled chicken breast (110 calories, 26g protein, 0g fiber, 25mg sodium, 200mg phosphorus, 10mg calcium, 250mg potassium)
  - 1/2 cup cooked quinoa (100 calories, 4g protein, 2g fiber, 5mg sodium, 80mg phosphorus, 10mg calcium, 150mg potassium)
  - 1 cup steamed broccoli (55 calories, 2g protein, 5g fiber, 55mg sodium, 60mg phosphorus, 70mg calcium, 450mg potassium)

Total: 265 calories, 32g protein, 7g fiber, 85mg sodium, 340mg phosphorus, 90mg calcium, 850mg potassium

**Snack**

- **Apple Slices with Almonds**
  - 1 medium apple (95 calories, 0g protein, 4g fiber, 0mg sodium, 10mg phosphorus, 10mg calcium, 150mg potassium)
  - 1 oz almonds (160 calories, 6g protein, 3g fiber, 0mg sodium, 130mg phosphorus, 75mg calcium, 200mg potassium)

Total: 255 calories, 6g protein, 7g fiber, 0mg sodium, 140mg phosphorus, 85mg calcium, 350mg potassium

**Dinner**

- **Baked Salmon with Brown Rice and Green Beans**
  - 3 oz baked salmon (180 calories, 20g protein, 0g fiber, 50mg sodium, 250mg phosphorus, 25mg calcium, 350mg potassium)
  - 1/2 cup cooked brown rice (110 calories, 2g protein, 1g fiber, 5mg sodium, 40mg phosphorus, 10mg calcium, 50mg potassium)
  - 1 cup green beans (55 calories, 2g protein, 2g fiber, 55mg sodium, 20mg phosphorus, 50mg calcium, 200mg potassium)

Total: 345 calories, 24g protein, 3g fiber, 110mg sodium, 310mg phosphorus, 85mg calcium, 600mg potassium

**Daily Totals**

- Calories: 1185
- Protein: 78g
- Fiber: 23g
- Sodium: 675mg
- Phosphorus: 1090mg
- Calcium: 605mg
- Potassium: 2370mg
- Fluids: Approximately 800mL from food, allowing for up to 400mL from drinks.

To meet your exact calorie and protein requirements, consider adding healthy fats like olive oil and nuts, and protein sources like lean meats, fish, eggs, and dairy products. Given your heart failure and diabetes, it's crucial to monitor your fluid intake closely and adjust the portion sizes based on your hunger and fullness cues.

**Budget Breakdown**

- Whole wheat bread: $0.50
- Eggs: $0.25
- Fresh berries: $1.00
- Yogurt: $0.50
- Chicken breast: $1.50
- Quinoa: $0.75
- Broccoli: $0.50
- Apple: $0.25
- Almonds: $1.00
- Salmon: $2.00
- Brown rice: $0.25
- Green beans: $0.50
  Total: $8.00

This plan should fit within your $12.50 daily budget, allowing for some flexibility in case you want to adjust the portions. Adjustments can be made to increase calories if needed. Also, consider consulting with your dietitian for personalized adjustments based on your health status and preferences.
